# Impacts of the Universal Credit welfare reform on well-being: a natural experiment study using UK population survey data

**DOI:** 10.1136/bmjph-2025-003762

**Published:** 2026-05-07

**Authors:** Andrew James Baxter, Martha Tindall, Sophie Wickham, Maria Marimpi, Heather Brown, Luke Munford, Matthew Sutton, Matteo Richiardi, Mandy Cheetham, Silas Amo-Agyei, David Taylor-Robinson, Clare Bambra, Srinivasa Vittal Katikireddi, Peter Craig

**Affiliations:** 1 School of Health and Wellbeing, University of Glasgow, Glasgow, UK; 2 Department of Public Health, Policy and Systems, University of Liverpool, Institute of Population Health, Liverpool, UK; 3 Division of Health Research, Lancaster University, Lancaster, UK; 4 Health Organisation, Policy and Economics, The University of Manchester, School of Health Sciences, Manchester, UK; 5 Centre for Microsimulation and Policy Analysis, University of Essex, Colchester, UK; 6 NIHR Applied Research Collaboration North East and North Cumbria, Gosforth, UK; 7 Department of Nursing, Midwifery and Health, Northumbria University, Newcastle upon Tyne, UK; 8 Population Health Sciences Institute, Newcastle University, Newcastle upon Tyne, England, UK

**Keywords:** Mental Health, Public Health, Sociodemographic Factors

## Abstract

**Introduction:**

Universal Credit (UC) was a large-scale reform of the UK welfare system, replacing six existing benefits. UC aimed to simplify claims and encourage more claimants into work. Previous research has found evidence of harm to the mental health of recipients, potentially exacerbating existing health inequalities. We identify the effect of UC on self-reported measures of psychological well-being, treating the phased rollout from 2013 to 2018 as a natural experiment.

**Methods:**

We estimated differences in psychological well-being outcomes associated with the staggered introduction of the UC across local authorities, using areas where UC was not yet available as controls. We included working-age (aged 18–64 years) respondents of the Annual Population Survey in Great Britain from 2012 to 2019 (n=245 658), living in low-income households. We used the four self-reported measures of psychological well-being recorded in the survey: Life Satisfaction, Happiness, Life Worthwhile and Anxiety. We tested for differential effects by disability, age, caring responsibilities, sex, country, ethnicity, education and household structure.

**Results:**

UC was associated with per-claimant decreases in Life Satisfaction (−0.66; 95% CI −1.01 to −‍0.30), Happiness (−‍0.41; 95% CI −‍0.77 to −0.05) and Life Worthwhile (−0.73; 95% CI −1.03 to −‍0.42), and increases in Anxiety (+0.79; 95% CI 0.30 to 1.27). These changes were 2–6 times as large as the effects on well-being of the COVID-19 pandemic. Respondents in Wales and Scotland saw comparatively greater effects compared with those in England across several outcomes. UC exposure saw greater comparative increases in anxiety among people with disabilities (+0.19; 95% CI 0.12 to 0.27), single people (+0.13; 95% CI 0.06 to 0.21) and people aged under 25 years (+0.27; 95% CI 0.15 to 0.39).

**Conclusions:**

The introduction of UC had adverse effects across all four measures of well-being. Vulnerable groups typically experienced greater harms, reinforcing calls for reforms to UC to reduce the health and well-being impacts of poverty and unemployment.

**WHAT IS ALREADY KNOWN ON THIS TOPIC**

- Universal Credit (UC)—a new benefits system—was introduced in the UK from 2013 to 2018.
- Changes to welfare policies may affect claimants’ mental health and well-being: UC has previously been shown to be detrimental to the mental health of claimants.

**WHAT THIS STUDY ADDS**

- The incremental rollout of UC led to large negative effects on well-being compared with areas that hadn’t yet undergone the change.
- Effects were greater among several vulnerable groups.

**HOW THIS STUDY MIGHT AFFECT RESEARCH, PRACTICE OR POLICY**

- Reforms to UC should prioritise the protection of the health and well-being of vulnerable households, addressing the potential harms of income insecurity and stringent conditions on benefit receipt.

## Introduction

Universal Credit (UC) was introduced in the UK under the 2012 Welfare Reform Act as a replacement for six existing working-age benefits and tax credits. The UC system was proposed as an innovation to simplify the benefits system and reduce spending.^1 2^ A further stated aim of UC was to encourage more claimants to work by setting stricter eligibility criteria and changing the payment structure.^2–4^ Welfare policies are an established determinant of health, and changes in social security systems are known to impact the mental health of benefit claimants.^5 6^ A simplified claims system, improved access to employment and a reduction in poverty have been proposed as routes to improving mental health and well-being via the UC system.^7 8^ Health commentators and researchers have expressed concerns about the design and implementation of UC and have called for clearer evaluation of potential health effects.^9–11^

Several studies on the implementation of UC suggest that it adversely affects the health and well-being of some recipients.^4 12–15^ Such harms may be a combination of the effects of switching to and navigating an unfamiliar online system, plus the lasting effects of a difference in award amounts or benefit administration. The minimum 5-week assessment period at the beginning of an award and administrative delays have resulted in waits of up to 12 weeks for first payments.^1 16^ This waiting period has been shown to cause immediate distress in low-income households.^16 17^ The subsequent struggle to repay loans and advance payments taken to cover the waiting period may also have prolonged this effect beyond the initial months.^18^ Fluctuations in income due to unstable employment and self-employment can lead to volatility and unpredictability in UC payments in the following months.^16 19^ Under 25s receive a lower monthly allowance than claimants aged 25 years or over; equivalent reductions were not applied to lone parents under previous benefits systems. More stringent work-search requirements combined with (the threat of) sanctions—reduced payments when conditions are not met—may also affect mental health and well-being throughout a spell of benefit receipt.^16 20^ The switch to a digital system, reported to be perceived by some claimants as ‘complicated, disorientating, impersonal, hostile and demeaning’,^16^ may also contribute to poorer mental well-being. Conversely, tailored support in applying for jobs may improve mental well-being through increased employment.^2 7^ Short-term effects of a new system may be reversed by such longer-term benefits. Effects may differ by family circumstances, reasons for claiming benefits and other health conditions, and UC has been shown to mitigate the negative impacts of entering unemployment for some claimants.^12 13^

The replacement of legacy benefits with UC has taken place in three phases. Each phase involved a staggered implementation across job centres.^1 2 21^ The first phase, beginning in 2013, was a restricted rollout of the ‘live service’ to a limited subgroup, mainly single, unemployed claimants without dependent children. In the second ‘natural migration’ phase, from 2015 onwards, new claimants, existing recipients whose circumstances changed and voluntary switchers moved onto UC. The concluding ‘managed migration’ phase, piloted in 2019–2020 but paused for the pandemic and restarted in 2022, involves a compulsory transition of all legacy claimants to UC. After repeated delays, this phase is currently planned for completion by 2028/2029.^22^ Wickham *et al*^4^ previously used the ‘restricted rollout’ phase to create comparable exposed and unexposed populations within the limited at-risk population defined by their employment status. The natural migration phase, now complete, gives an opportunity to the test effects across the broader scope of all eligible claimants over a longer period to test whether early observed harms persist or are ameliorated by the benefits of a maturing and adapting new system.

We aimed to estimate the effect of introducing UC on the well-being of working-age individuals in low-income households over the full natural migration rollout period. Self-reported measures of psychological well-being are valuable for understanding the effects of welfare reform on mental health and how they vary among population subgroups.^23–26^ Evidence on changes in population well-being is increasingly recommended to inform economic policy.^27^ Estimates of the changes in subjective well-being resulting from the UC rollout would provide a tangible measure of its overall impact on those affected by the benefit system reform.

We used the staggered rollout of the natural migration phases to create natural experimental comparisons between people living in areas exposed and areas not yet exposed to UC. To better understand impacts on inequalities, we investigated how effect sizes varied by characteristics that may determine eligibility and award amounts or that may make an individual more vulnerable to changes: family structure, sex, disability, ethnicity, age, education, student status, caring responsibilities and country. We hypothesised that exposure to UC would reduce well-being among claimants and that greater effects would be seen among vulnerable populations.

## Methods

We followed a prepublished protocol^28^ and analysis plan.^29^ Deviations from the analysis plan are outlined in online supplemental F.

10.1136/bmjph-2025-003762.supp1 Supplementary data

### Study design

**Figure 1:**
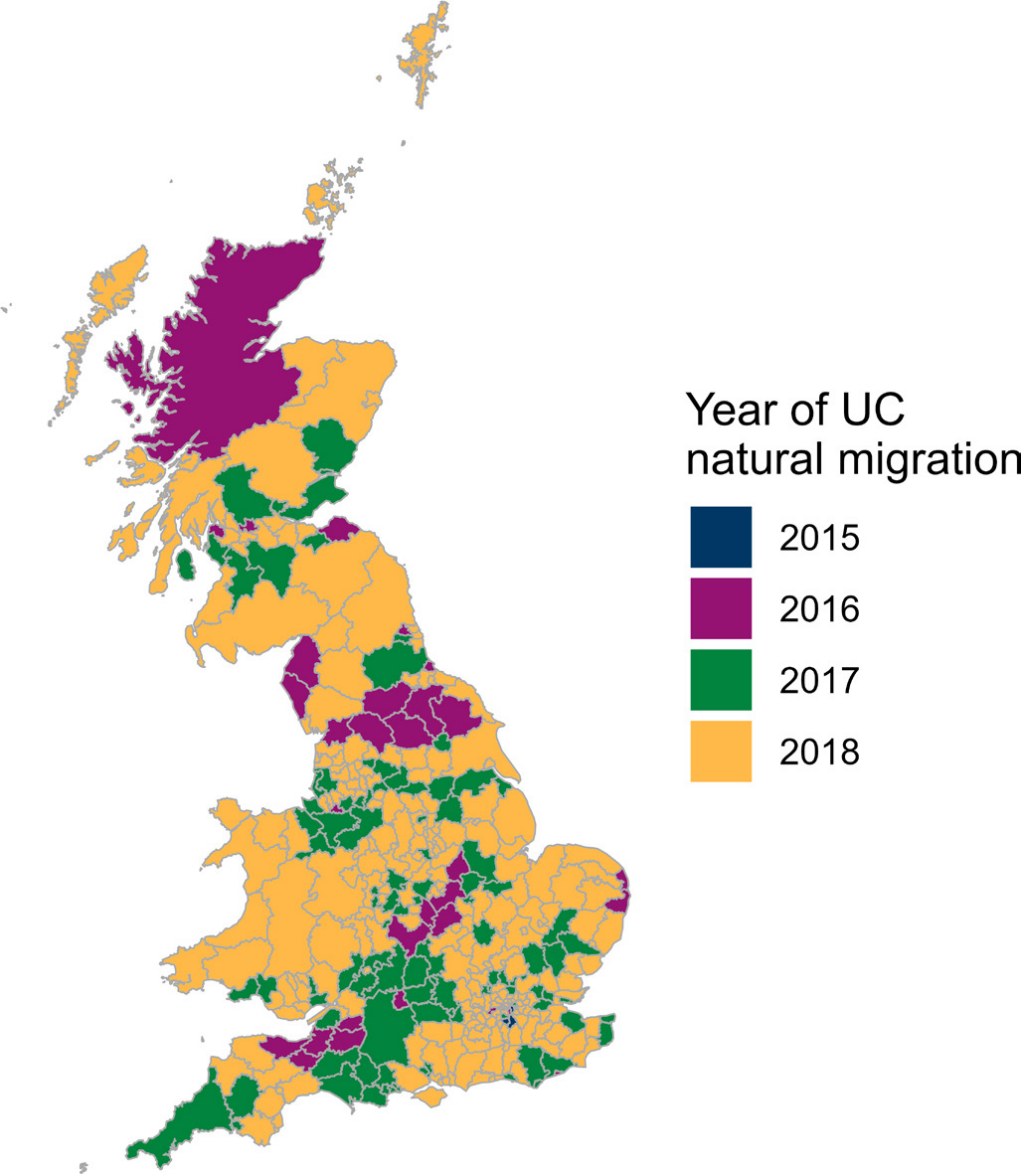
Yearly Universal Credit (UC) rollout by local authority (LA) area. Dates for each Jobcentre area are extracted from the Department for Work and Pensions schedule^21^ and grouped by LA.

We conducted a difference-in-differences analysis to estimate the effects of the staggered rollout of UC on well-being among recipients and potential recipients. We examined changes over time in four well-being measures—Life Satisfaction, Happiness, Life Worthwhile and Anxiety—in local authorities as UC was introduced. We compared these with simultaneous changes in areas in which it had not yet been introduced to account for common trends. We took an intention-to-treat approach, using the planned natural migration dates as a proxy for whole-area exposure.^21^ Previous studies found no association between tested demographic variables (ethnicity, labour market attachment, marital status and health) and rollout date.^12^ Consultations with the study advisory committee and representatives from the Department for Work and Pensions indicated that decisions regarding order of rollout were determined by administrative reasons and prioritised geographic spread—these decisions were not based on performance indicators or relating to expected outcomes. Thus, the order of rollout by local authority (LA) appears sufficiently random to treat this staggered pattern of exposure as a natural experiment (figure 1).

### Data

We used data from Annual Population Surveys (APS) collected from April 2012 to March 2020 (April–March pooled datasets, 921 139 observations).^30^ Previous and simultaneous research on the effects of UC has used the UK Household Longitudinal Survey and measures of mental health, quality of life and well-being.^4 12 13 15 28^ We sought to complement this research using a large, cross-sectional dataset and further well-being measures. The Annual Population Survey is a collation of the Labour Force Survey responses, consecutively gathered across five quarters. Only respondents’ quarters one and five responses are included in sequential annual APS datasets, which are weighted to be representative cross-sections of the UK population. The survey records details of employment and benefit receipt (by type), alongside demographic variables.^31^ Questions recording personal well-being across four measures—Life Satisfaction, Happiness, Life Worthwhile and Anxiety—were introduced from April 2012.^32^ We conducted a complete case analysis across all APS respondents in the 2012–2020 period, as data across all selected variables were missing in only 1.6% of cases (14 745 observations removed).

We used the Office for National Statistics (ONS) personal well-being variables as measures of outcome and so included data from April 2012 onwards. We excluded observations from 2020 onwards, as these would include COVID-19-affected responses and may have produced different patterns of employment and benefit claims. We used a secure access version of the dataset in which each respondent’s area of residence was recorded, allowing for grouping of observations by LA district. These districts were mapped onto the Department of Work and Pensions’ Jobcentre areas that were used in the ‘natural migration’ rollout of UC from 2015 to 2018.^21^ We excluded Local Authorities with low numbers of observations (fewer than 100) in any quarter (8920 observations removed).

To control for area-level confounders, we calculated yearly proportional changes in economic productivity and local government spending from the 2012 baseline. We used data from the ONS ‘Regional economic activity by gross domestic product’ dataset to calculate gross value added to represent yearly economic productivity by UK LA.^33^ To represent local government spending, we used recorded spending per capita on social care, culture and education, as these were deemed not to be directly affected by UC rollout. These were obtained for England from the ‘Place-based Longitudinal Data Resource’,^34–36^ StatsWales open data for Welsh LAs^37^ and from Scottish Local Government Finance Statistics (collated across years on email request).^38^

### Population

To identify an ‘at-risk’ population comparable across all time points, we included residents of low-income households, aged 18 years or over, who were not retired and not working 20 or more hours per week. This would include most respondents who are eligible for benefits but may also include some who are not eligible. We use income rather than reported benefit receipt to identify the at-risk population for two reasons: (1) benefit receipt may have been under-reported in the survey, and (2) the effects of the change from legacy benefits to UC may extend beyond benefit recipients, for example, by prompting claimants to enter or increase employment and end their benefit claim. By including all low-income households, we aimed to make all observations comparable across time periods.

To identify low-income households, we calculated household equivalised income using the modified Organisation for Economic Co-operation and Development (OECD) equivalence scale.^39^ We set a threshold of under £12 000 equivalised annual household income for inclusion. This was derived from the upper quartile of incomes among earners reporting benefit claims and the lower quartile of earners reporting no benefit claims (online supplemental A,table A3 and figure A1).

In sensitivity analyses, we repeated these analyses for all respondents reporting a benefit claim (UC or at least one equivalent legacy benefit) and for higher and lower wage thresholds based on median and 90^th^ income percentiles to test for similarities in effect direction and magnitude.

### Exposure

We used the planned rollout dates to determine exposure to UC.^21^ Dates were available from Jobcentre Plus, which, in most cases were grouped by LA. This level of geography allowed matching of observations to rollout dates and the use of area-level covariates. We matched APS observations by LA and calendar quarter to assign a dummy variable coding UC exposure (‘1’ in the quarter of rollout and all subsequent quarters; ‘0’ otherwise) and a count of quarterly leads/lags to UC rollout (centred at ‘0’ in the rollout quarter).

### Outcomes

We used the ‘ONS4’ personal well-being measures to capture the impact of the UC rollout on individuals’ well-being and lived experiences across four key domains: Life Satisfaction, Happiness, Life Worthwhile and Anxiety. Respondents were asked to rate how they were currently feeling in each domain from 0 to 10. For Life Satisfaction, Happiness and Life Worthwhile, 0 represented the lowest level of well-being and 10 the highest. For anxiety the scale is reversed (see questions in online supplemental A,section A.2). Unlike traditional economic and social metrics, subjective well-being reflects individuals’ lived experiences, preferences and personal values, capturing the net impact of policy changes on diverse groups. These measures offer a multidimensional view of well-being by assessing Life Satisfaction, emotional state and a sense of meaning and purpose in life—critical for understanding the nuanced effects of welfare reform on individuals’ quality of life.^25^ By treating these outcomes as continuous variables,^32^ we can quantify the specific ways the UC rollout influenced well-being, providing valuable insights into the reform’s overall impact on vulnerable populations.

### Covariates

In confounder-adjusted models, estimates were adjusted for age, age squared, sex, ethnicity (combined into two categories: white and non-white), disability, whether the respondent has a work-limiting health condition, highest level of qualification, employment status (employed/inactive/seeking), housing tenure, whether the respondent has caring responsibilities (reporting not seeking work as looking after home/family), number of children (categorised 0, 1 or 2+), marital status (non-married or married/cohabiting), year of observation, area-level unemployment rate, area-level disability rate, area-level gross value added, area-level culture spending per capita (adjusted relative to 2013) and quarter of LA migration to UC.

### Statistical analysis

We used difference-in-differences methods to estimate the effects of the introduction of UC on well-being. We used person weights as provided in the APS datasets to make the sample representative of the UK population by sex and age group.^40^

Classic difference-in-differences models examining staggered exposure across multiple units use two-way fixed-effects (TWFE) models to estimate average effects of exposure. This method assumes that treatment effects do not change over time: if treatment leads to changes in trends, early-treated units become controls for later-treated units, thus potentially biasing results.^41–43^ This assumption is likely to be violated in examining the effects of UC due to incremental increases in the number of claimants and thus an intensifying population-wide effect in the post-rollout period.

To study differences in the effect across the post-rollout period robustly, we used several methods to account for this expected bias.^42^ We used the two-stage difference-in-differences method and the ‘did2s’ R package^43 44^ as the most suitable for our (non-balanced) data. This method estimates a ‘never-treated’ potential outcome from a regression across not-yet-treated observations while accounting for period and time effects before estimating effects as observed differences from this imputed counterfactual outcome. Each model estimated dynamic event-study estimates for each outcome across quarters following rollout and a static effect estimate across the whole exposed period. We recorded static estimates—average effects across the exposure period—as primary measurements of effect size for interpretability. We plotted event-study estimates to visually examine changes over time. In our main analysis, we included all observations across the 2012–2019 period.

As a sensitivity analysis, we fitted a model to a ‘truncated’ dataset to exclude observations relatively distant from the rollout date. We excluded early observations before 2013 and late observations more than 2 years after UC rollout, relative to respondents’ local area.

We conducted TWFE sensitivity analyses to test whether the expected biases produced differing estimates, as outlined in our protocol.^29^ We fitted unadjusted regressions and fully adjusted TWFE models, limiting post-rollout observations to the four quarters following the rollout date in each LA. We fitted two further adjusted models with one post-rollout time point at 1 year and 2 years after the natural migration date—this prevents the use of post-rollout observations as controls and estimates time-changing effects.

To give interpretable estimates of effects, population-wide differences in outcomes were scaled to a ‘per claimant’ indicator of effect sizes by dividing the estimated average change in outcome by the proportion of respondents reporting UC receipt in the exposed period. Per-claimant standardised measures of change from pre-rollout means were calculated by dividing scaled estimates by pre-rollout SDs. To place the effect estimates in context, we used the April 2020–March 2021 APS dataset to estimate corresponding per-person effects of entering the COVID-19 pandemic across all UK households.

To test for differences of effects on vulnerable populations, we estimated effects for the following subgroups: full-time students, young people (aged under 25 years, matching standard allowance threshold),^45^ people with disabilities, people with dependent children, single people, people with caring responsibilities, lone parents, women, people of non-white ethnicity and people with lower education levels. We also compared Scotland and Wales with England to test for differences in effects across the three countries which may be produced by differences in benefit administration.

We tested for parallel pre-intervention trends across all LA areas grouped by quarter of UC rollout (13 quarters). We plotted quarterly pre-rollout mean outcomes across all grouped observations with fitted trend lines and inspected these visually. We further tested differences in trends relative to the trends of the latest quarter for unadjusted and fully adjusted models.

All statistical analyses were conducted in R (V.4.2.2).^46^ Rendered documents containing analysis results were added to the Open Science Framework archive (OSF) at osf.io/knajb.^29^

## Results

We included 245 658 observations in our study sample (table 1). Using survey weighting for representativeness, the mean age was 39.2 years in pre-local-rollout periods and 39.9 years in post-rollout periods exposed to UC. 55.2% of pre-rollout and 56.4% of post-rollout respondents were women. Most respondents were white (80.9% pre-rollout and 78.6% post-rollout), living in England (85.2% and 86.5%), not disabled (65.5% and 61.9%) and living in rented accommodation (63.2% in both periods). Pre-rollout respondents had a mean Life Satisfaction score of 7.0 (SD=2.1), mean Happiness score of 7.0 (SD=2.4), mean Life Worthwhile score of 7.4 (SD=2.1) and mean Anxiety score of 3.4 (SD=3.0). Each outcome increased by ~0.1 points after rollout (not adjusting for pre-rollout trends).

**Table 1:** Population demographics, counted by observation and summarised by survey weighting

Demographic	Pre-rollout	Post-rollout
Observed population		
Unweighted (N)	193 668	51 990
Weighted (%)	76.5	23.5
Well-being outcomes—mean (SD)		
Life Satisfaction	7.0 (2.1)	7.1 (2.1)
Happiness	7.0 (2.4)	7.1 (2.4)
Life Worthwhile	7.4 (2.0)	7.5 (2.1)
Anxiety	3.4 (3.0)	3.5 (3.1)
Age		
Mean (SD)	39.2 (14.3)	39.9 (14.4)
Sex (N (%))		
Male	74 214 (44.8)	19 477 (43.6)
Female	119 454 (55.2)	32 513 (56.4)
Ethnicity (N (%))		
White	166 152 (80.9)	44 171 (78.6)
Non-white	27 516 (19.1)	7819 (21.4)
Country (N (%))		
England	144 110 (85.2)	38 982 (86.5)
Wales	24 175 (5.7)	5447 (4.8)
Scotland	25 383 (9.1)	7561 (8.7)
Disabled (N (%))		
Not disabled	116 526 (65.5)	29 256 (61.9)
Disabled	77 142 (34.5)	22 734 (38.1)
Work-limiting health condition (N (%))		
No	126 539 (70.3)	32 193 (67.5)
Yes	67 129 (29.7)	19 797 (32.5)
Highest level of qualification (N (%))		
Degree or college	48 300 (25.7)	14 805 (28.6)
Upper secondary	43 053 (25.9)	11 688 (26.8)
Lower secondary	47 215 (23.1)	12 283 (21.8)
Tertiary	23 250 (11.6)	5427 (10.2)
None	31 850 (13.7)	7787 (12.5)
Employment status (N (%))		
In employment	82 480 (41.8)	22 659 (43.4)
ILO unemployed*	21 963 (12.8)	4231 (9.1)
Inactive	89 225 (45.5)	25 100 (47.5)
Housing tenure (N (%))		
Outright	40 252 (16.9)	12 023 (18.4)
Mortgaged	41 292 (18.8)	10 012 (17.1)
Rented	110 378 (63.2)	29 413 (63.2)
Other	1746 (1.1)	542 (1.3)
Relationship status (N (%))		
Married/cohabiting/civil partnership	93 404 (43.0)	24 613 (42.1)
Non-married	100 264 (57.0)	27 377 (57.9)
Number of children (N (%))		
0	120 235 (61.5)	33 617 (64.0)
1	32 495 (17.4)	7736 (15.2)
2+	40 938 (21.1)	10 637 (20.8)
Caring responsibilities (N (%))		
No	161 672 (84.7)	43 670 (85.4)
Yes	31 996 (15.3)	8320 (14.6)

Means, percentages and SDs are weighted for representativeness. Total unweighted n=245 658.

*Classed as ‘unemployed’ under the International Labour Organisation (ILO) definition—not currently working in reference period, able to start work within two weeks and currently seeking work.

N, unweighted counts of observations.

An average of 6.3% of the sample reported receiving UC in the first year after rollout, increasing to 9.4% across all post-rollout time periods (37.8% of respondents reported still claiming legacy benefits in the post-rollout period). Small proportions (<2%) of respondents reported claiming UC in the period before the ‘natural migration’ rollout date (see online supplemental figure A2), potentially having transitioned to UC during the ‘restricted rollout’ phase. A clear change in trend is seen at the intended transition period, with the number of UC-claiming respondents rising rapidly in the first four quarters after exposure and continuing beyond this period. There is a rapid decrease in the number of observations in later quarters, with only 67 of the 380 LAs being observed for 9 or more quarters post-rollout.

**Figure 2:**
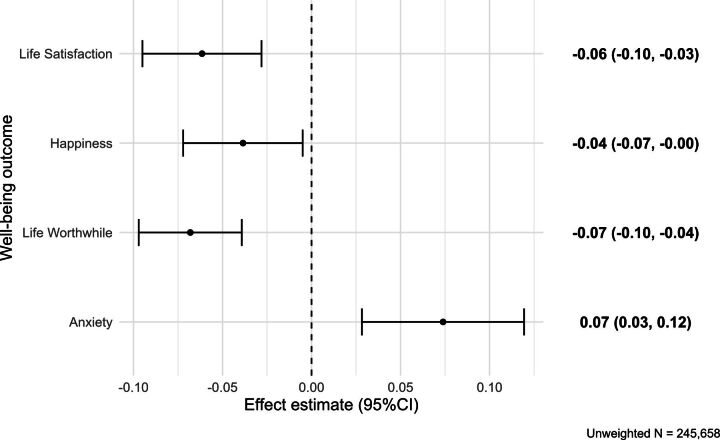
Well-being effects across all observed post-rollout periods, estimated using a 'two-stage difference-in-differences’ model adjusted for all confounders.

Two-stage model estimates are presented in table 2 and figure 2. In adjusted models, rollout of UC was associated with an average −0.06 point drop in Life Satisfaction (95% CI −0.10 to −0.03), a −0.04 point drop in Happiness (95% CI −0.07 to −0.00), a −0.07 point drop in life rated as ‘worthwhile’ (95% CI −0.10 to −0.04) and a 0.07 point increase in Anxiety (95% CI 0.03 to 0.12; figure 2). Given that on average 9.4% of exposed people report claiming UC, this is equivalent to a −0.66 (95% CI −1.01 to −0.30) point per claimant change in Life Satisfaction (−0.31 SDs from a pre-rollout mean of 7.0), a −0.41 (95% CI −0.77 to −0.05) point per claimant change in Happiness (−0.17 SDs from a mean of 7.0), a −0.73 (95% CI −1.03 to −0.42) point per claimant change in feeling life is worthwhile (−0.36 SDs from a mean of 7.4) and a 0.79 (95% CI 0.30 to 1.27) point per claimant change in Anxiety (0.26 SDs from a mean of 3.4; table 2). To put these effects in context, the impact on psychological well-being across all survey respondents of entering the COVID-19 pandemic was smaller in each of the four domains (table 2).

**Table 2:** Estimated effects of UC from difference-in-differences models

Outcome	Two-stage difference-in-differences estimates		Per-person effect	
	Unadjusted	Fully adjusted	Switching to UC	Entering COVID-19 pandemic*
Life Satisfaction	−0.077 (−0.125 to −0.029)	−0.062 (−0.095 to −0.028)	−0.66 (−1.01 to −0.30)	−0.23 (−0.30 to −0.17)
Happiness	−0.059 (−0.109 to −0.010)	−0.039 (−0.072 to −0.005)	−0.41 (−0.77 to −0.05)	−0.23 (−0.36 to −0.11)
Life Worthwhile	−0.076 (−0.121 to −0.032)	−0.068 (−0.097 to −0.039)	−0.73 (−1.03 to −0.42)	−0.12 (−0.16 to −0.08)
Anxiety	0.099 (0.033 to 0.165)	0.074 (0.028 to 0.119)	+0.79 (0.30 to 1.27)	+0.43 (0.20 to 0.67)

Adjusted models are scaled up to per-person estimates and compared with the effect of the COVID-19 pandemic.

All estimates are shown with 95% CIs in brackets.

*‘COVID-19 pandemic’ effects estimated as a weighted average, adjusting for year trends and month of observation; all survey participants. These estimates are not related to changes to UC claims during the pandemic.

UC, Universal Credit.

**Figure 3:**
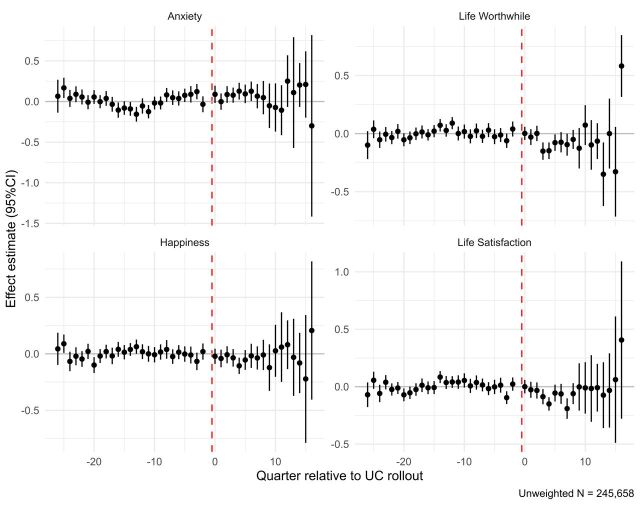
Dynamic event-study plots of effects by quarter relative to the rollout of Universal Credit (UC) (at quarter ‘0’).

In event-study plots produced from two-stage difference-in-differences models, across all four outcomes, we see indicators of harm across the early period after the rollout dates (around the first 2 years), with diverging and uncertain effect estimates and effect directions in later periods (figure 3). Over the first eight quarters, we observed small, mostly negative effects, with CIs not including zeros in a few cases: for Life Satisfaction we saw significant effects in three periods (third, fourth and seventh quarters after rollout), for Happiness we saw effects in only one period (fourth quarter), for Life Worthwhile we saw effects in three periods (third, fourth and fifth quarters) and for Anxiety in two periods (fourth and sixth quarters).

In subgroup analyses, we found evidence of greater effects across several markers of vulnerability (online supplemental C). Single people saw greater effects on Life Satisfaction and Life Worthwhile and greater Anxiety (online supplemental C and figure C1). People with disabilities experienced greater effects across all four well-being domains (online supplemental C and figure C2). We found larger effects of the UC rollout on Anxiety among people aged under 25 years, women, carers and full-time students, with no clear differences across other outcomes. Few clear differences in effect were seen across levels of educational attainment (online supplemental figure C10). We found no clear evidence of UC-related effects on well-being among people from minority ethnic groups, while people of white ethnicity experienced negative effects across all four outcomes (online supplemental figure C9).

Across the three countries, there was some evidence of larger adverse effects among people in Wales on Life Satisfaction (−0.07 (95% CI −0.14 to −0.01)), Happiness (−0.11 (95% CI −0.22 to −0.01)), Life Worthwhile (−0.07 (95% CI −0.16 to 0.02)) and Anxiety (0.12 (95% CI −0.07 to 0.31)) than people in England. Scottish respondents saw greater harms to Life Satisfaction (−0.09 (95% CI −0.16 to −0.02)) compared with England (online supplemental table C1 and figure C7).

Across all observations, parents with caring responsibilities for 1+ children saw more positive outcomes in Life Satisfaction (0.12 (95% CI 0.07 to 0.18)), Happiness (0.11 (95% CI 0.04 to 0.18)) and Life Worthwhile (0.08 (95% CI 0.03 to 0.13)) compared with non-parents. Lone parents experienced greater harm to Life Satisfaction (−0.06 (95% CI −0.14 to 0.02)) and Anxiety (0.20 (95% CI 0.03 to 0.31)) compared with coupled parents, with the largest increases in anxiety across all groups, while seeing potentially positive differences in the other outcomes from single non-parents.

Across pre-intervention periods, grouped by quarter of rollout, most groups show stable trends in Anxiety and small increases across Happiness, Life Satisfaction and Life Worthwhile, with some diverging trends (online supplemental figure A3). Once adjusting for potential confounders, any differential trends across LAs were largely eliminated (online supplemental figure A5). We took these conditional parallel trends in the pre-rollout period as satisfying the parallel trends assumption. We hypothesised that anticipatory effects would largely be independent of rollout dates, as most claimants were likely unaware of UC coming to their own Jobcentre next month. To test for anticipatory effects, we visually examined differences in outcomes in the quarters preceding rollout in event-study plots and found no consistent indicators of anticipatory effects.

### Sensitivity analyses

Two-way fixed effects estimates are presented in Supplement B. Across the first year following UC rollout, estimated effect directions and magnitudes are very similar to two-stage model estimates (online supplemental figure B9). Effects observed at the single time point 1 year after rollout were larger than period-average effects (2–3 times larger than first-year averages). At 2 years, effects were reduced across all four outcomes, with large CIs likely driven by smaller numbers of observations (online supplemental figure B10).

In truncated models, restricting to observations from 2013 until 2 years after rollout dates, effect estimates were similar to main analyses for Life Satisfaction (−0.06 (95% CI −0.09 to −0.03)), Happiness (−0.04 (95% CI −0.07 to −0.00)) and Life Worthwhile (−0.06 (95% CI −0.09 to −0.03)) and smaller effects were seen for Anxiety (0.03 (95% CI −0.01 to 0.08); online supplemental figure B12).

Restricting analyses to 189 844 respondents reporting a benefit claim (UC or one of the six ‘legacy benefits’) produced larger effect estimates for Life Satisfaction (−0.10 (95% CI −0.14 to −0.06)), Happiness (−0.07 (95% CI −0.11 to −0.03)), Life Worthwhile (−0.10 (95% CI −0.13 to −0.07)) and Anxiety (0.10 (95% CI 0.04 to 0.15); online supplemental D). Reported receipt of UC was greater in this population (18.1% compared with 9.8% of the low-income population, which includes non-claimants). These estimates scale to a per-claimant effect on Life Satisfaction of −0.54 (95% CI −0.76 to −0.33), Happiness of −0.38 (95% CI −0.60 to −0.15), Life Worthwhile of −0.56 (95% CI −0.73 to −0.39) and Anxiety of 0.54 (95% CI 0.25 to 0.82).

Setting equivalised income thresholds for inclusion to £8040 per year (the median value of UC recipients’ incomes) decreased the number of observations to 222 492, of which 9.9% reported claiming UC in post-rollout years. Estimates of outcomes were similar in magnitude to the main analysis outcomes (online supplemental figure E2). Increasing the threshold to the 90th percentile value of £18 600 increased observations to 444 069, of which 6.2% reported claiming UC. Again, outcomes remained similar to the main analyses (online supplemental figure E4). These tests indicate that outcomes were not sensitive to the chosen threshold of £12 000.

## Discussion

During the restricted rollout and natural migration phases from 2013 to 19, implementation of UC was associated with a reduction in each of the measured domains of well-being among adults in low-income households. These effects persisted across the first 2 years of rollout in each locality and were consistent across models with differing assumptions. We identified variation in the effects of UC among specific subpopulations, notably greater anxiety among young people, people with disabilities, women, full-time students, those with caring responsibilities and single people (with a stronger effect on lone parents). Conversely, people of non-white ethnicity experience fewer adverse effects than those of white ethnicity, and couples with children may have experienced improved well-being. People living in Scotland and Wales experienced poorer outcomes in some domains compared with people living in England. The potentially greater impact in Scotland is contrary to the expected effects of differences in the administration of benefits, which aimed to reduce the impact of benefits system change; these estimates may indicate that early amendments were ineffective or insufficient.^47^ Similar effects in Scotland and Wales may be indicative of populations that were more vulnerable to the UC rollout. The Scottish Child Payment, introduced in 2021 after the observation period of this study, awarded additional amounts to UC claimants with children to reduce child poverty^48^ and may mitigate some of these effects in Scotland.

The effect sizes across the population represent an average effect in the magnitude of ~0.1 points on the 10-point scale but are potentially produced by a comparatively small subset of the observed population, with fewer than 10% of respondents reporting UC receipt. When standardising to a per-person effect (under the assumption that the effects of benefits change were fully or substantially felt by those switching to the new benefit), these represent potentially substantial impacts on a person’s well-being. Although small in absolute terms, they are considerably larger (1.8 to over 6 times) than the effects of the COVID-19 pandemic (table 2).

Well-being measures provide valuable insights into the complex effects of policy changes. Public consultations identified ‘Life satisfaction’ as an important indicator of national well-being, and it is increasingly used in economic analyses through the WELLBY (well-being adjusted life year) framework to quantify and monetise the well-being effects of policies.^25 27^ In our baseline analysis, the estimated 0.66-point reduction in Life Satisfaction across the UC rollout period translates into a significant well-being cost. Based on the recommended WELLBY values of £10 000 to £16 000 per person per year,^27^ this reduction corresponds to a monetary loss of between £6600 and £10 560 per person per year (in 2019 prices). These figures highlight the substantial welfare costs of the rollout of the policy, which should be considered in further economic evaluations of the reform and in the costs of future changes to welfare.

Our findings are consistent with previous research, which finds harms to mental health associated with the transition to UC and additional harms for some groups of claimants.^5 15 49 50^ Our recently published analysis of harms to mental health, conducted using longitudinal data from Understanding Society, found similar estimates of harm.^15^ In this analysis, we were able to observe a larger population and estimate differential effects of subgroups while examining well-being outcomes as complementary measures. Our observations build on Wickham *et al*’s^4^ analysis of changes across the ‘restricted rollout’ period by assessing effects across all low-income households, including both employed and unemployed potential UC claimants, during the subsequent (‘natural migration’) phase of UC implementation. Our findings on well-being follow a similar pattern to earlier reports of unequal increases in psychological distress. Exploring similar axes of inequality as examined by Brewer *et al*,^12^ we find lower well-being among single people and lone parents. These observations are consistent with the finding of poorer mental health of such groups entering unemployment under UC relative to the legacy benefits. Similar to Thornton and Iacoella,^13^ we found smaller effects on claimants with children than those without. The exception to this pattern appears to be the increase in Anxiety among lone parents over all other family structures. Proposed positive aspects of UC—if indeed effective, for example, simplifying the claims process or incentivising entry into work—may have produced stronger effects in some claimants, or social circumstances may have buffered some of the negative effects felt more strongly by others.

We were unable to distinguish the immediate effects of transitioning to or claiming UC from the lasting effects of the difference in benefit administration or payment amounts. Our data examined the population cross-sectionally. An observed increase in the proportions of respondents reporting claiming UC, as expected from the structure of the ‘natural migration’ rollout, would produce greater average effects across time if the treatment effect were static. Some indication of intensifying effects can be seen across the initial quarters of the exposed period, which is consistent with this. Alternatively, if a more intense ‘shock’ effect were seen by individuals on first switching, this effect would reduce over time. As data did not record the length of time on UC, we were unable to test for individual-level dynamics of effects.

Our analyses are limited to the period 2013–2019, so we were not able to assess the effects of the two-child limit introduced in 2017. Likewise, we did not examine the effectiveness of pandemic-related changes to UC, as these came after the natural migration rollout phase. Our analyses assumed an ‘as if’ random rollout of UC by LA in the absence of a formal outline of how the order of rollout was decided.^51^ Clustering of areas with similar factors affecting well-being earlier or later in the rollout schedule may have introduced bias if these corresponded with either other national events or changes in UC implementation. Testing of pre-UC trends across all LAs, grouped by rollout quarter, showed no evidence of diverging trends, which strengthens the inference that subsequent differences are produced by the transition to UC. Further analyses using more granular markers of geographically determined exposure, where available, may allow more precise identification of exposed units.

Our outcomes of Life Satisfaction, Life Worthwhile, Happiness and Anxiety were selected as measures available in the dataset. These well-being outcomes, while validated measures used in other analyses of population-level mental health and well-being, do not translate directly into clinical outcomes or tangible life experiences. Our comparison to pandemic changes and monetising of WELLBY outcomes aims to give relatable contexts for these estimates.

Another limitation of our study is that our comparison across low-income households had low specificity in identifying claimants. Our use of an income marker would also have excluded higher-income households with circumstances eligible for higher extra award amounts.^45^ The potential insensitivity of using observed benefit receipt and the threat to exchangeability of exposed and unexposed populations are discussed above. Using an equivalised income threshold determined from the data, our intention-to-treat approach aimed to include all households who were ‘at risk’ from changes in benefit systems, exchangeable across exposure states. Similarities in effect estimates from analyses restricted to those reporting benefit receipt suggest that our low-income household estimates are generalisable to the benefit-claiming population. Other approaches, such as propensity score matching, could be applied to compare units reporting UC receipt with comparable units in unexposed areas to achieve specificity while maintaining exchangeability.

### Implications

Our results add to existing evidence that the rollout of UC has had adverse effects on the well-being of claimants and potential claimants. Greater award amounts, a more accessible claims procedure and more flexible application of conditionality could mitigate these effects.^5^ Further investigation should seek to identify which of the hypothesised mechanisms are most important, particularly for vulnerable households, which have been shown to be more adversely affected and should therefore be the focus of efforts to improve the design and delivery of UC.

## Data Availability

Data may be obtained from a third party and are not publicly available. Participant survey data are available through the Secure Access service at UK Data Service. The following datasets were used:Office for National Statistics, Social Survey Division. Annual Population Survey, 2004-2022: Secure Access. [data collection]. 29th Edition. UK Data Service, 2023 [Accessed 18 June 2024]. Available from: DOI: http://doi.org/10.5255/UKDA-SN-6721-28. Office for National Statistics, Social Survey Division. Annual Population Survey Household, 2004-2021: Secure Access. [data collection]. 9th Edition. UK Data Service, 2023 [Accessed 18 June 2024]. Available from: DOI: http://doi.org/10.5255/UKDA-SN-6725-9.
