# Supplementary material for: Impacts of the Universal Credit welfare reform on well-being: a natural experiment study using UK population survey data
Source: BMJ Public Health. 2026 May 7;4(2):e003762. doi: 10.1136/bmjph-2025-003762 (PMC13158600; doi:10.1136/bmjph-2025-003762)
Supplement: Supplementary data [file bmjph-4-2-s001.pdf]

Supplement A - Selecting population and pre-analysis checks

A.1 Population counts and data removed due to missingness or ineligibility.

Level	Removed	Population
All observations	-	921,139
No missing data	14,745	906,394
Sufficient numbers of observations in area	8,920	898,104
Over 18 and pre-2020	83,372	814,787
Low-income population	569,129	245,658

Table A-1 – Population inclusion by criteria

A.2 Outcome variable questions

The wellbeing outcomes are captured in the 'ONS 4' questions on wellbeing: 'Life satisfaction', 'Happiness', 'Life worthwhile' and 'Anxiety' (Table A-2).

Question wording	Scale	Variable name in dataset
Overall, how satisfied are you with your life nowadays?	0 = 'not at all satisfied'<br>To<br>10 = 'completely satisfied'	SATIS
How happy did you feel yesterday?	0 = 'not at all happy'<br>To<br>10 = 'completely happy'	HAPPY
Overall, to what extent do you feel that the things you do in your life are worthwhile?	0 = 'not at all worthwhile'<br>To<br>10 = 'completely worthwhile'	WORTH

Question wording	Scale	Variable name in dataset
How anxious did you feel yesterday?	0 = 'not at all anxious'<br>To<br>10 = 'completely anxious'	ANXIOUS

Table A-2 – Questions, scales and variable names of the four wellbeing questions asked of APS respondents

A.3 Income across populations

	Lower quartile		Median		Upper quartile	
**Received UC**	**Income**	**Unweighted N**	**Income**	**Unweighted N**	**Income**	**Unweighted N**
No	£1,100	1,906	£1,830	2,866	£2,730	728
Yes	£390	1,584	£670	3,569	£1,070	2,066

Unweighted N = 542,383

Table A-3 – Median, lower quartile and upper quartile of monthly equivalised income across households receiving and not receiving Universal Credit or a legacy benefit

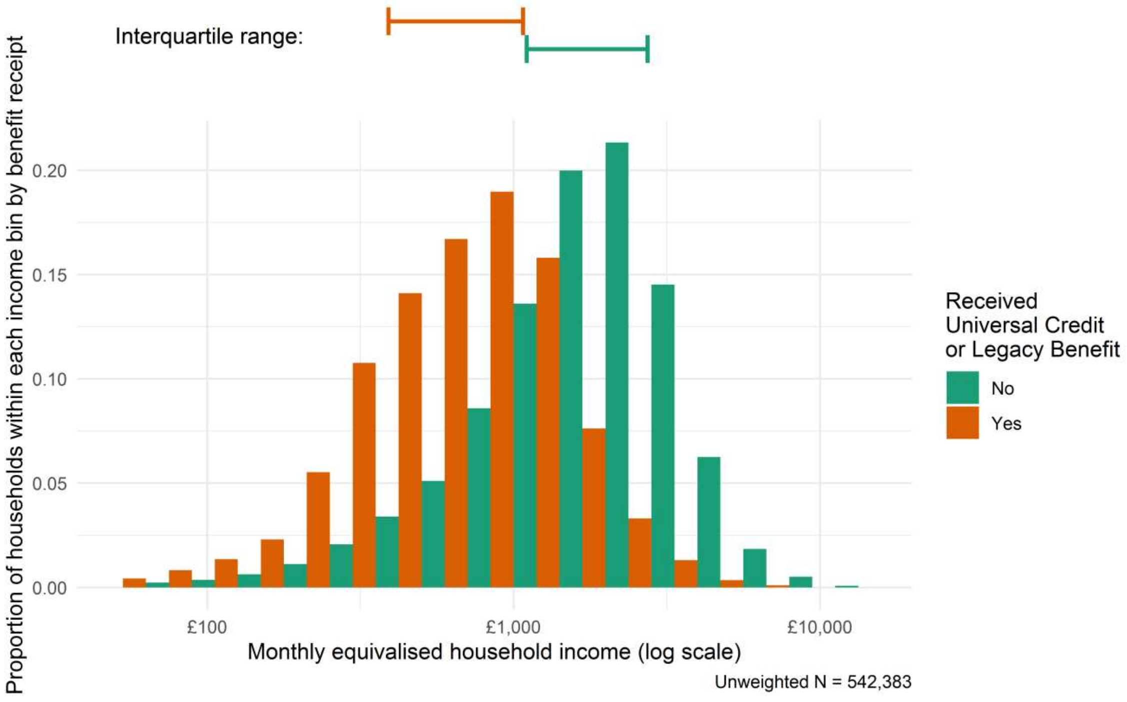

Figure A-1 – Distribution of Household equivalised income across reported receipt of UC or a Legacy Benefit, bars with fewer than 50 observations removed.

A.4 Exposure in post-UC period

Population = 245,658 (unweighted individuals). Weighted observations: 88,122,688 - living in households with equivalised household income < £12,000pa, not retired and not working more than average 20 hours per week.

Figure A-2 shows the percentage of low-income respondents in each period relative to the rollout of UC who report claiming Universal Credit.

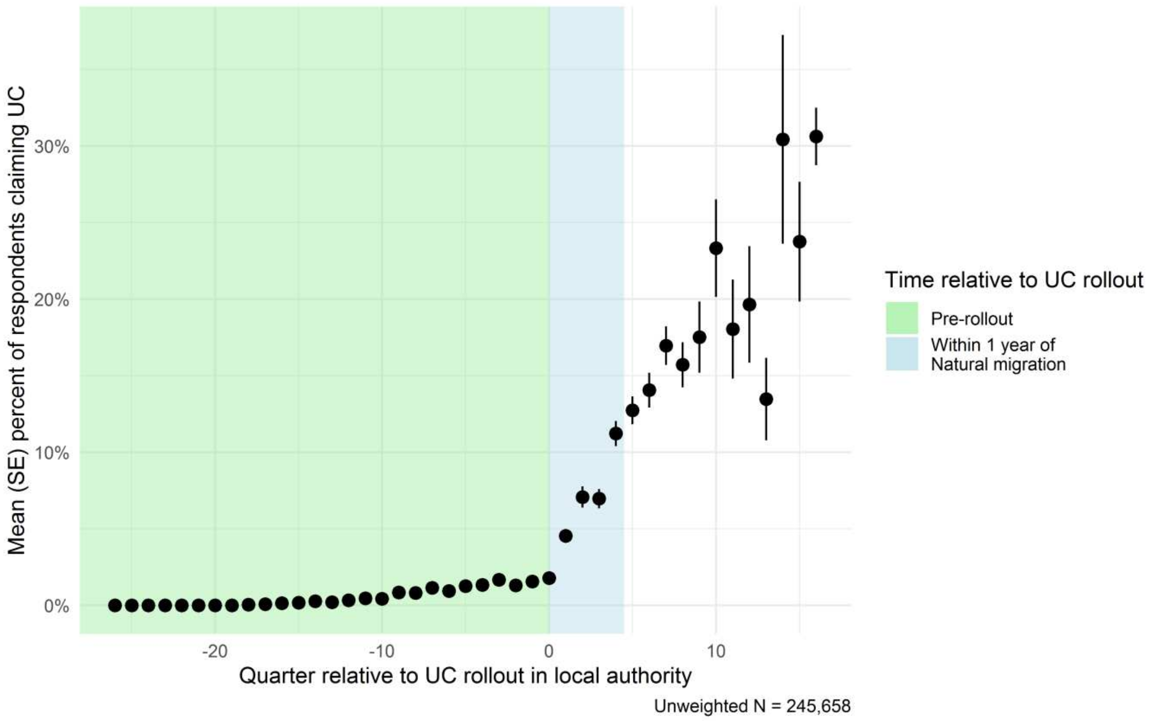

Figure A-2 – Percentage of respondents per Local Authority reporting claiming UC in each quarter relative to UC rollout in their area.

A.5 Low income population within 1 year

An average of 6.3% (N = 2089) of respondents in low-income households reported receiving Universal Credit across the first year following UC rollout.

9.4% (N = 4666) reported receiving UC across all years where UC was available in their Local Authority (37.8%, N = 22520 claimed legacy benefits).

At 1 year, 10.6% (N = 695) of surveyed respondents in low-income households reported receiving UC. At 2 years, 15.7% (N = 370) reported receiving UC.

A.6 Trends before intervention

Figure A-3 shows fitted trends across outcomes in the period before UC rollout. Figure A-4 shows the unadjusted differences between pre-intervention trends across grouped rollout periods. Figure A-5 shows the differences in trends when correcting for covariates.

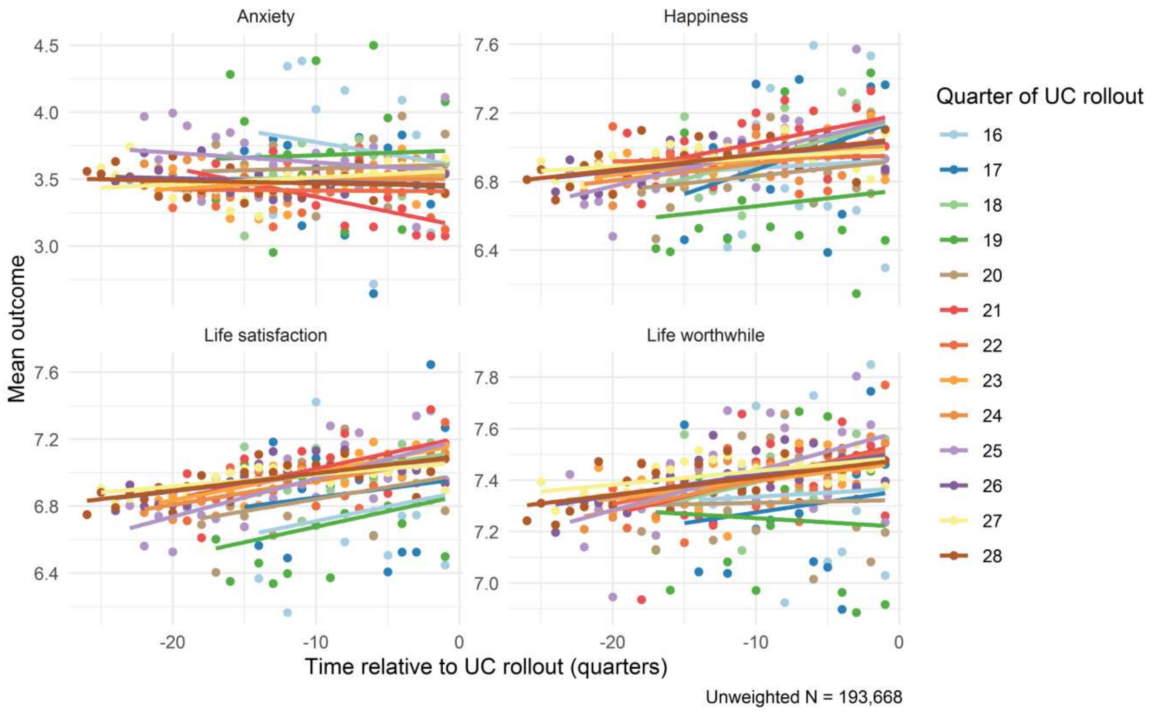

Figure A-3 – Trends in anxiety, life worthwhile, life satisfaction and happiness in pre-UC period, grouped by quarter of rollout for low income households. Dots represent mean scores for each outcome across all respondents grouped by quarter of rollout.

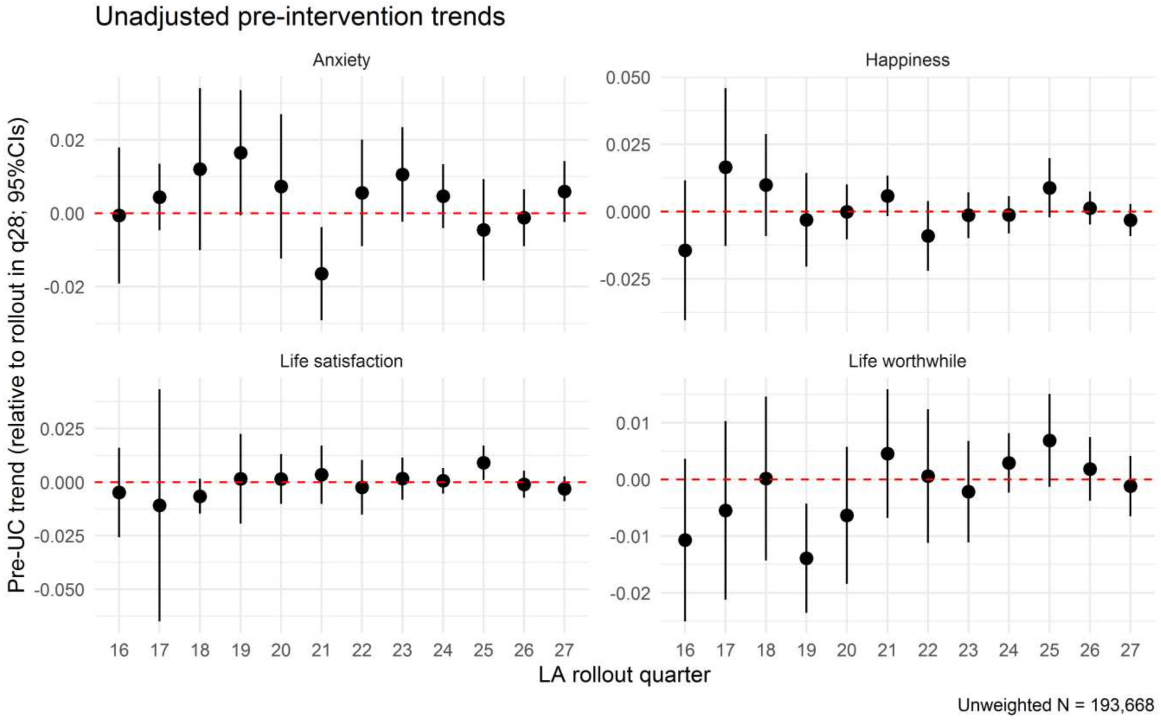

Figure A-4 – Differences in pre-exposure trends in anxiety, happiness, life satisfaction and life worthwhile. Areas grouped by quarter of rollout, relative to latest quarter (Q28 = Q4, 2018).

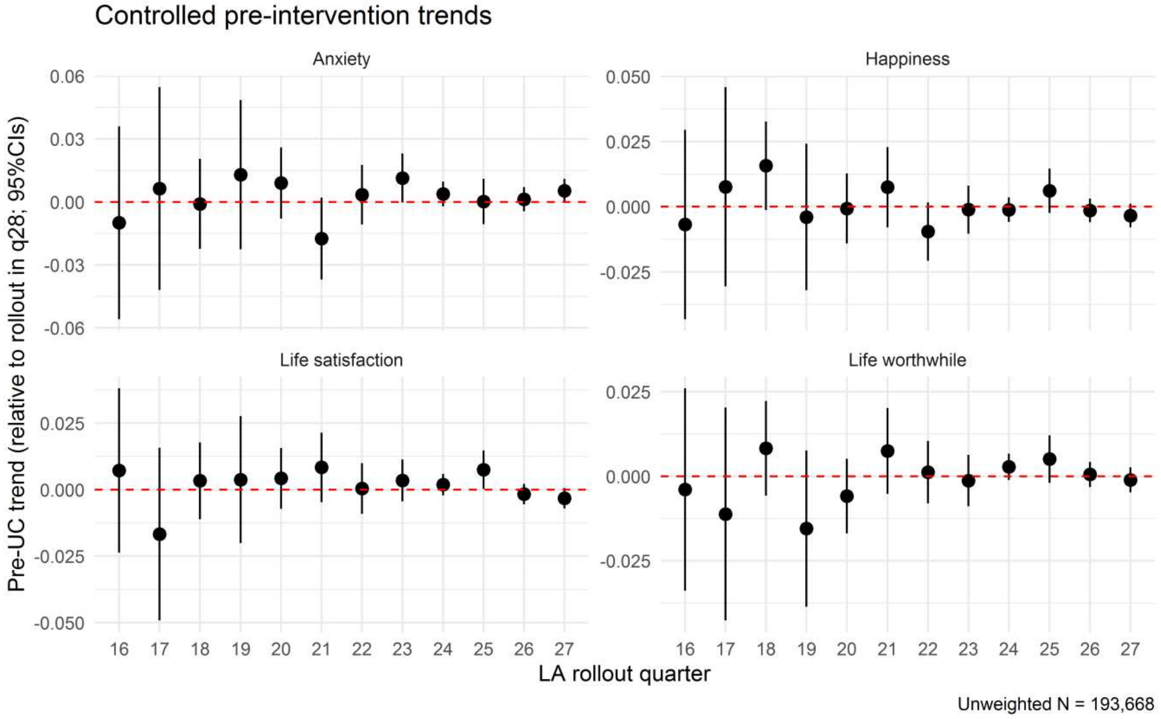

Figure A-5 – Differences in pre-exposure trends in anxiety, happiness, life satisfaction and life worthwhile, adjusted for all controls. Areas grouped by quarter of rollout, relative to latest quarter (Q28 = Q4, 2018).

Supplement B - Main effects analysis outputs

B.1 Life Satisfaction

B.1.1 TWFE models

B.1.1.1 In first year of rollout

Table B-1: Unadjusted and fully adjusted differences in 'Life Satisfaction' across 1st year

	Unadjusted regression		Fully adjusted TWFE	
**term**	**Estimate**	**95%CI**	**Estimate**	**95%CI**
UC rollout				
	0.0886	0.0652, 0.1120	-0.0489	-0.0899, -0.0079

Unweighted N = 245,658

B.1.1.2 Observations at 1-year and 2-years

Table B-2: Adjusted differences in 'Life Satisfaction at 1 year and 2 years post-UC

	1 year post-UC		2 years post-UC	
**term**	**Estimate**	**95%CI**	**Estimate**	**95%CI**
UC rollout				
	-0.1483	-0.2126, -0.0841	-0.0630	-0.1579, 0.0319

Unweighted N = 245,658

B.1.2 Two-stage models

B.1.2.1 All observations

Table B-3: Static effects of UC on Life Satisfaction (Two-stage model)

	Unadjusted			Fully Adjusted		
**Coefficient**	**Estimate**	**Standard Error**	**95%CI**	**Estimate**	**Standard Error**	**95%CI**
UC exposure	-0.0769	0.0245	-0.1248, -0.0290	-0.0615	0.0171	-0.0950, -0.0281

Unweighted N = 245,658

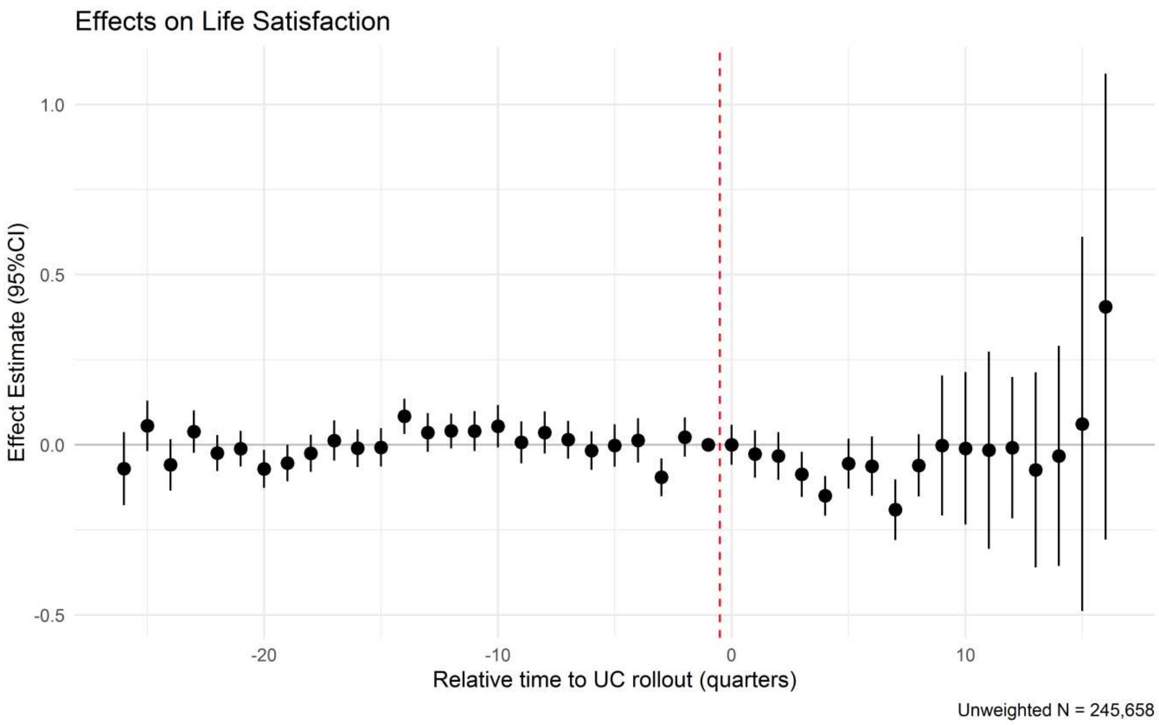

Figure B-1 Event-study plot of two-stage DiD Life Satisfaction model

B.1.2.2 Truncated time period

Table B-4: Static effects of UC on Life Satisfaction (Two-stage model)

Coefficient	Estimate	Standard Error	95%CI
UC exposure	-0.0578	0.0152	-0.0876, -0.0280

Unweighted N = 213,829

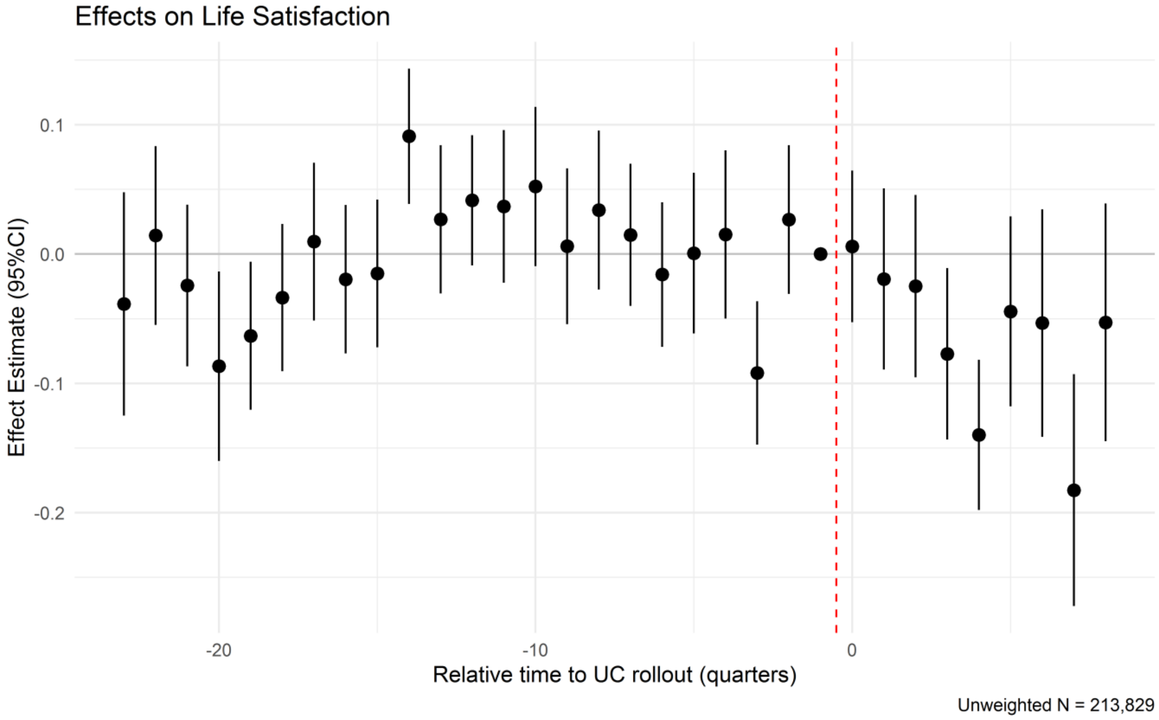

Figure B-2 Event-study plot of two-stage DiD Life Satisfaction model across time-truncated population

B.2 Happiness

B.2.1 TWFE models

B.2.1.1 In first year of rollout

Table B-5: Unadjusted and fully adjusted differences in ‘Happiness’ across 1st year

	**Unadjusted regression**		**Fully adjusted TWFE**	
**term**	**Estimate**	**95%CI**	**Estimate**	**95%CI**
UC rollout				
	0.0804	0.0537, 0.1070	-0.0393	-0.0855, 0.0070

Unweighted N = 245,658

B.2.1.2 Observations at 1-year and 2-years

Table B-6: Adjusted differences in ‘Happiness at 1 year and 2 years post-UC

	**1 year post-UC**		**2 years post-UC**	
**term**	**Estimate**	**95%CI**	**Estimate**	**95%CI**
UC rollout				
	-0.1108	-0.1862, -0.0355	-0.0172	-0.1515, 0.1172

Unweighted N = 245,658

B.2.2 Two-stage models

B.2.2.1 All observations

Table B-7: Static effects of UC on Happiness (Two-stage model)

Coefficient	Unadjusted Estimate	Standard Error	95%CI	Fully Adjusted Estimate	Standard Error	95%CI
UC exposure	-0.0594	0.0253	-0.1089, -0.0099	-0.0385	0.0171	-0.0721, -0.0050

Unweighted N = 245,658

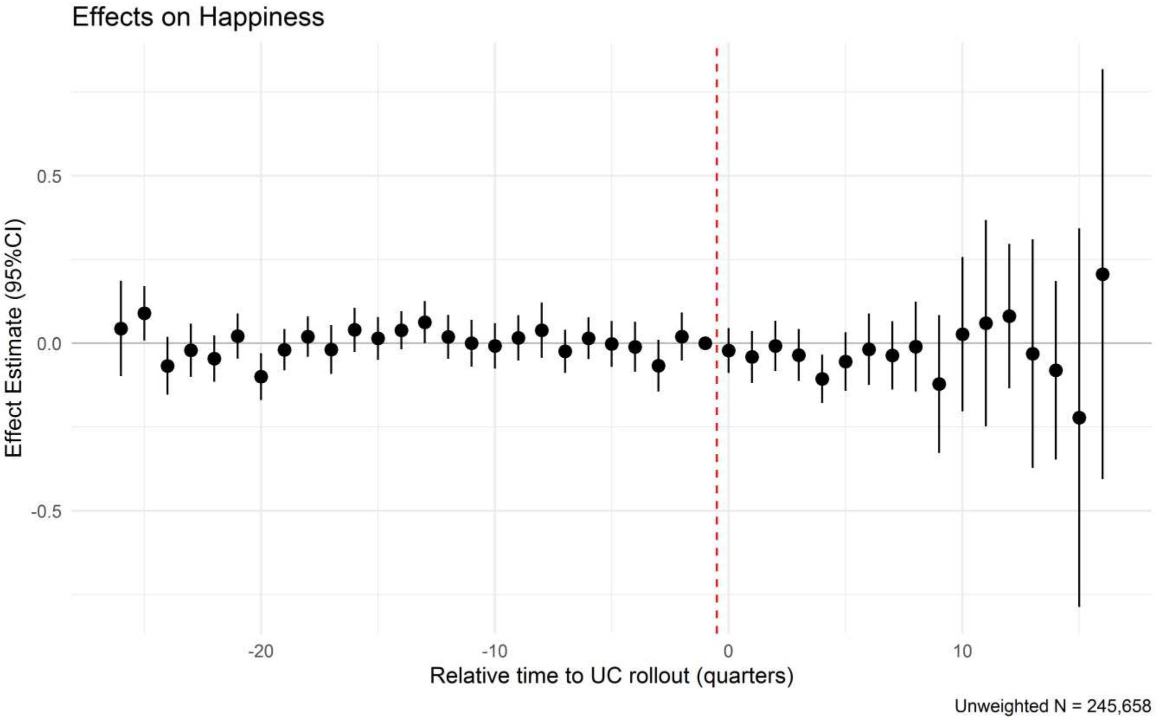

Figure B-3 Event-study plot of two-stage DiD Happiness model

B.2.2.2 Truncated time period

Table B-8: Static effects of UC on Happiness (Two-stage model)

Coefficient	Estimate	Standard Error	95%CI
UC exposure	-0.0360	0.0168	-0.0689, -0.0031

Unweighted N = 213,829

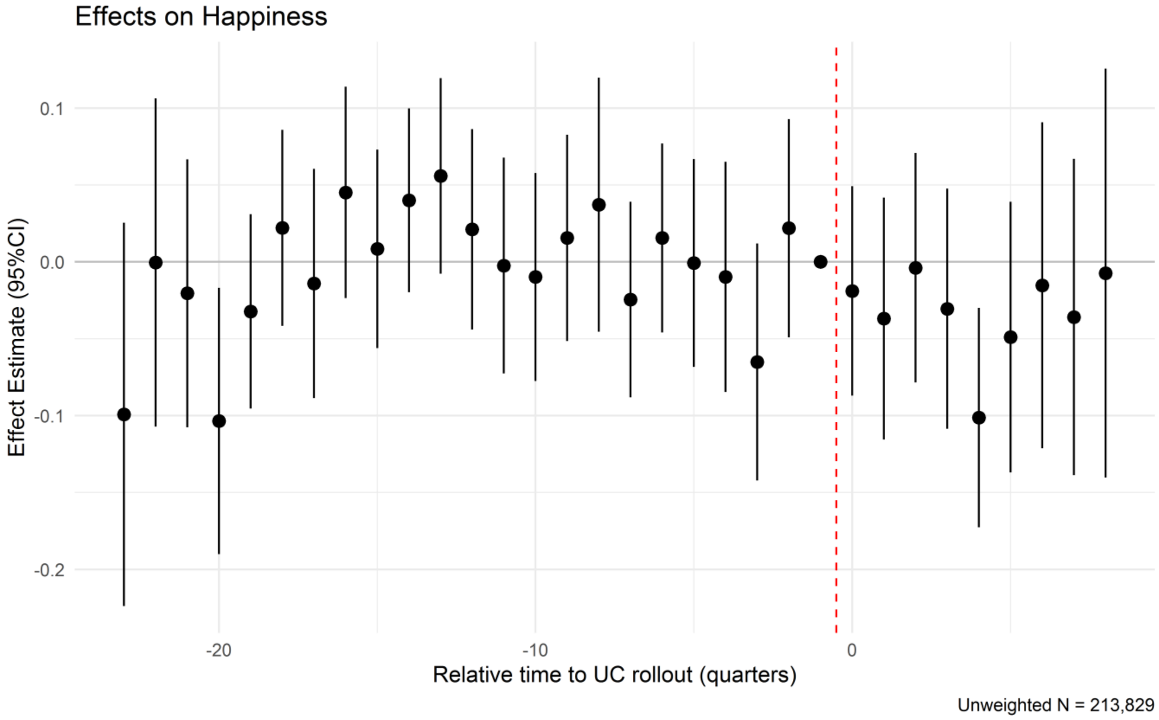

Figure B-4 Event-study plot of two-stage DiD Happiness model across time-truncated population

B.3 Life Worthwhile

B.3.1 TWFE models

B.3.1.1 In first year of rollout

Table B-9: Unadjusted and fully adjusted differences in ‘Life Worthwhile’ across 1st year

	Unadjusted regression		Fully adjusted TWFE	
**term**	**Estimate**	**95%CI**	**Estimate**	**95%CI**
UC rollout				
	0.0535	0.0311, 0.0760	-0.0607	-0.1026, -0.0188

Unweighted N = 245,658

B.3.1.2 Observations at 1-year and 2-years

Table B-10: Adjusted differences in ‘Life Worthwhile at 1 year and 2 years post-UC

	1 year post-UC		2 years post-UC	
**term**	**Estimate**	**95%CI**	**Estimate**	**95%CI**
UC rollout				
	-0.1507	-0.2261, -0.0754	-0.0548	-0.1411, 0.0315

Unweighted N = 245,658

B.3.2 Two-stage models

B.3.2.1 All observations

Table B-11: Static effects of UC on Life Worthwhile (Two-stage model)

	Unadjusted			Fully Adjusted		
**Coefficient**	**Estimate**	**Standard Error**	**95%CI**	**Estimate**	**Standard Error**	**95%CI**
UC exposure	-0.0761	0.0227	-0.1207, -0.0315	-0.0681	0.0148	-0.0971, -0.0392

Unweighted N = 245,658

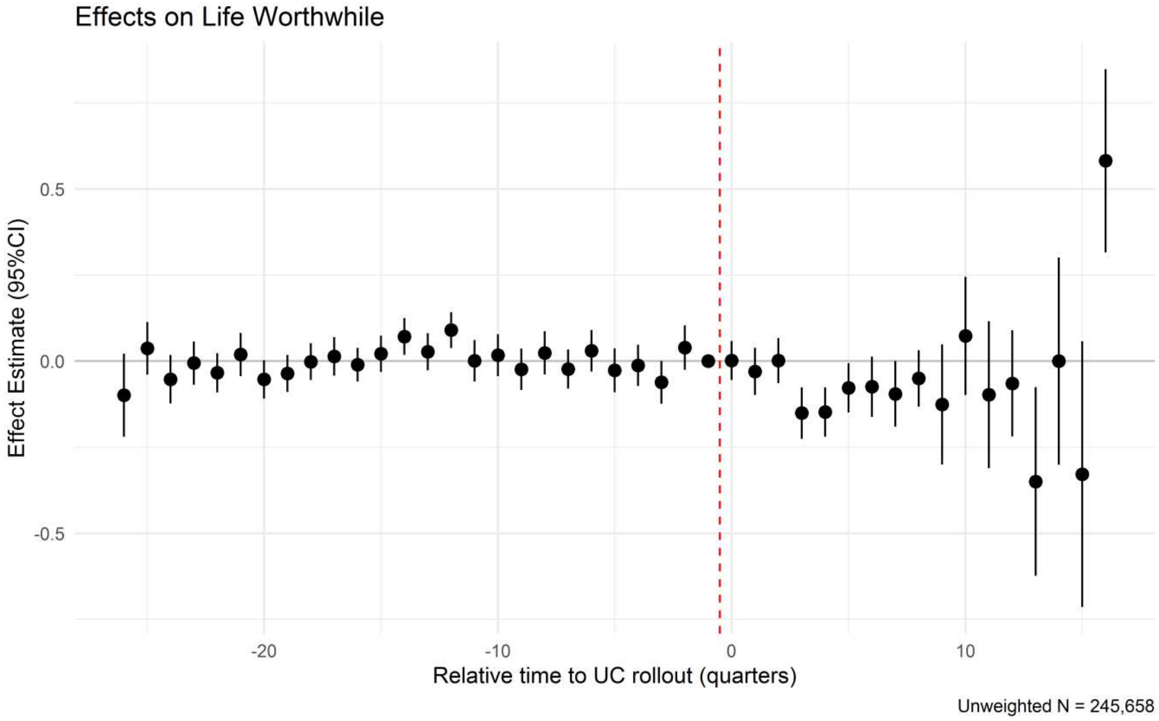

Figure B-5 Event-study plot of two-stage DiD Life Worthwhile model

B.3.2.2 Truncated time period

Table B-12: Static effects of UC on Life Worthwhile (Two-stage model)

Coefficient	Estimate	Standard Error	95%CI
UC exposure	-0.0559	0.0154	-0.0861, -0.0257

Unweighted N = 213,829

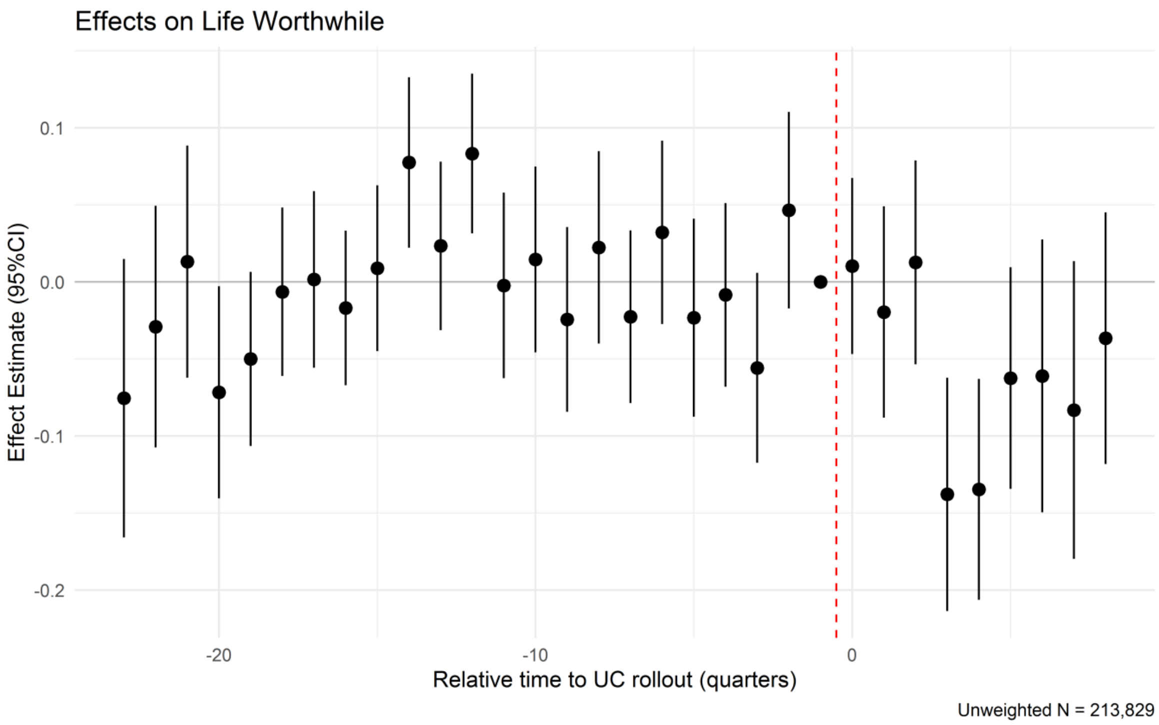

Figure B-6 Event-study plot of two-stage DiD Life Worthwhile model across time-truncated population

B.4 Anxiety

B.4.1 TWFE models

B.4.1.1 In first year of rollout

Table B-13: Unadjusted and fully adjusted differences in ‘Anxiety’ across 1st year

	**Unadjusted regression**		**Fully adjusted TWFE**	
**term**	**Estimate**	**95%CI**	**Estimate**	**95%CI**
UC rollout				
	0.1014	0.0679, 0.1350	0.0646	0.0015, 0.1278

Unweighted N = 245,658

B.4.1.2 Observations at 1-year and 2-years

Table B-14: Adjusted differences in ‘Anxiety at 1 year and 2 years post-UC

	**1 year post-UC**		**2 years post-UC**	
**term**	**Estimate**	**95%CI**	**Estimate**	**95%CI**
UC rollout				
	0.1213	0.0206, 0.2220	0.0467	-0.1604, 0.2538

Unweighted N = 245,658

B.4.2 Two-stage models

B.4.2.1 All observations

Table B-15: Static effects of UC on Anxiety (Two-stage model)

	**Unadjusted**			**Fully Adjusted**		
**Coefficient**	**Estimate**	**Standard Error**	**95%CI**	**Estimate**	**Standard Error**	**95%CI**
UC exposure	0.0989	0.0335	0.0333, 0.1645	0.0739	0.0232	0.0283, 0.1194

Unweighted N = 245,658

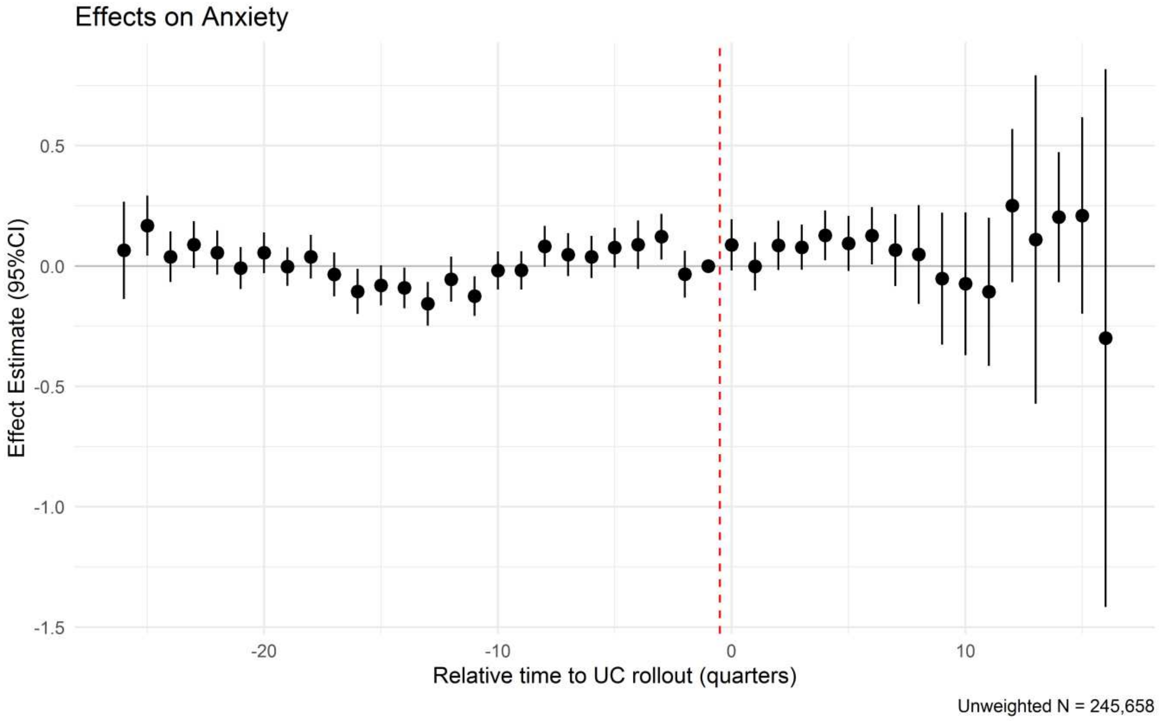

Figure B-7 Event-study plot of two-stage DiD Anxiety model

B.4.2.2 Truncated time period

Table B-16: Static effects of UC on Anxiety (Two-stage model)

**Coefficient**	**Estimate**	**Standard Error**	**95%CI**
UC exposure	0.0330	0.0237	-0.0135, 0.0795

Unweighted N = 213,829

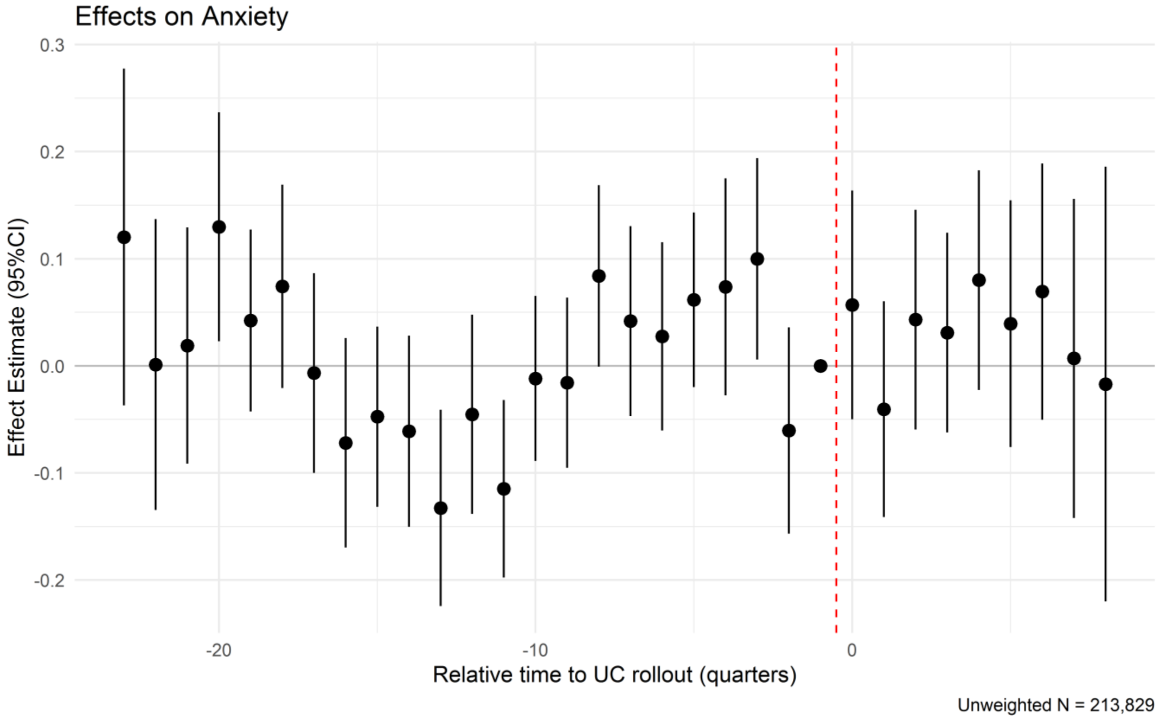

Figure B-8 Event-study plot of two-stage DiD Anxiety model across time-truncated population

B.5 Model summaries

B.5.1 1st year TWFE summary

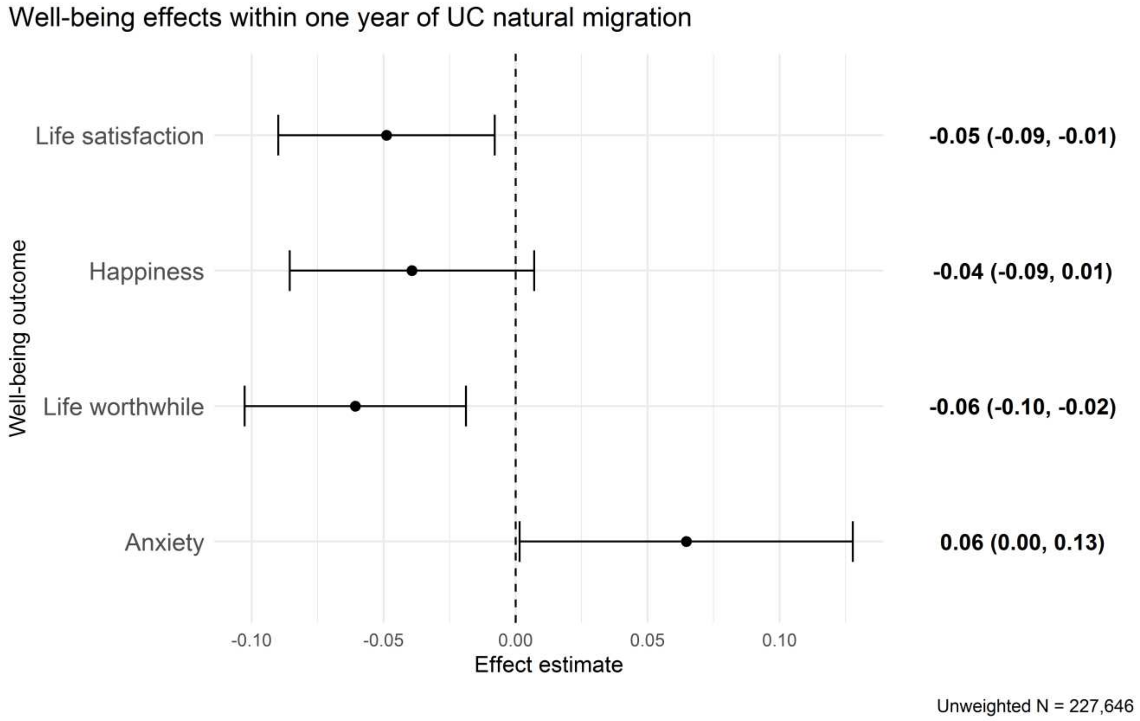

Figure B-9 Well-being effects within one year of UC natural migration (two-way fixed effects model)

B.5.2 1 vs 2 years TWFE

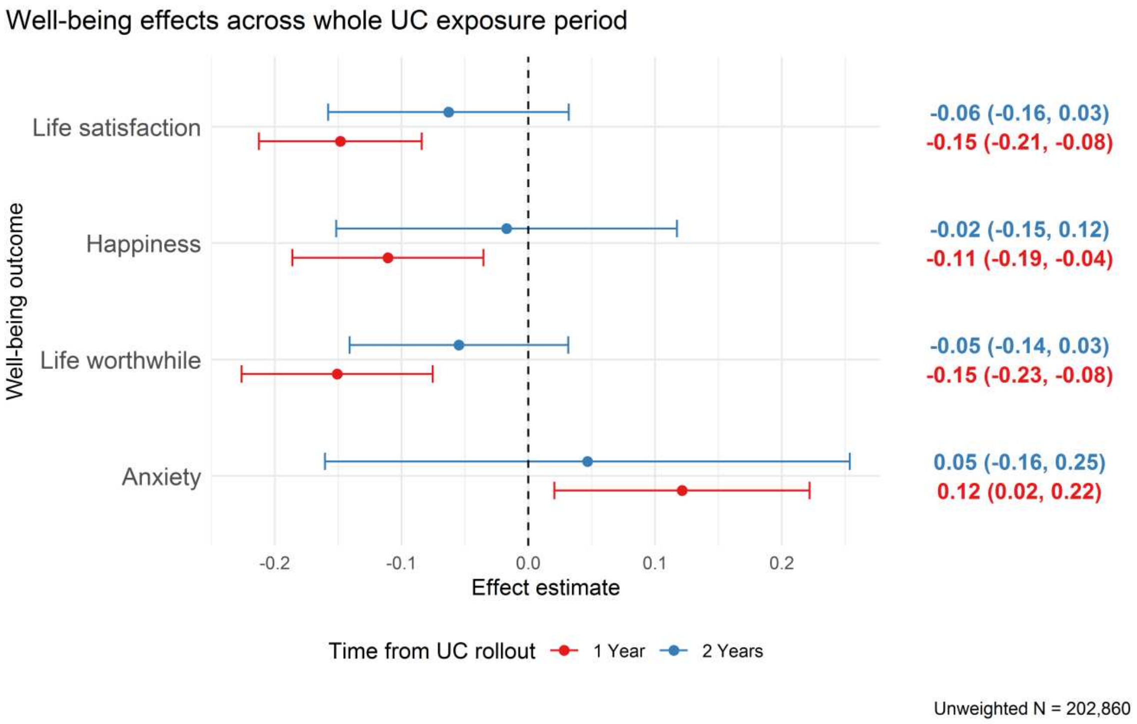

Figure B-10 Well-being effects in population - observations 1 year and 2 years after rollout

B.5.3 Two-stage DiD models

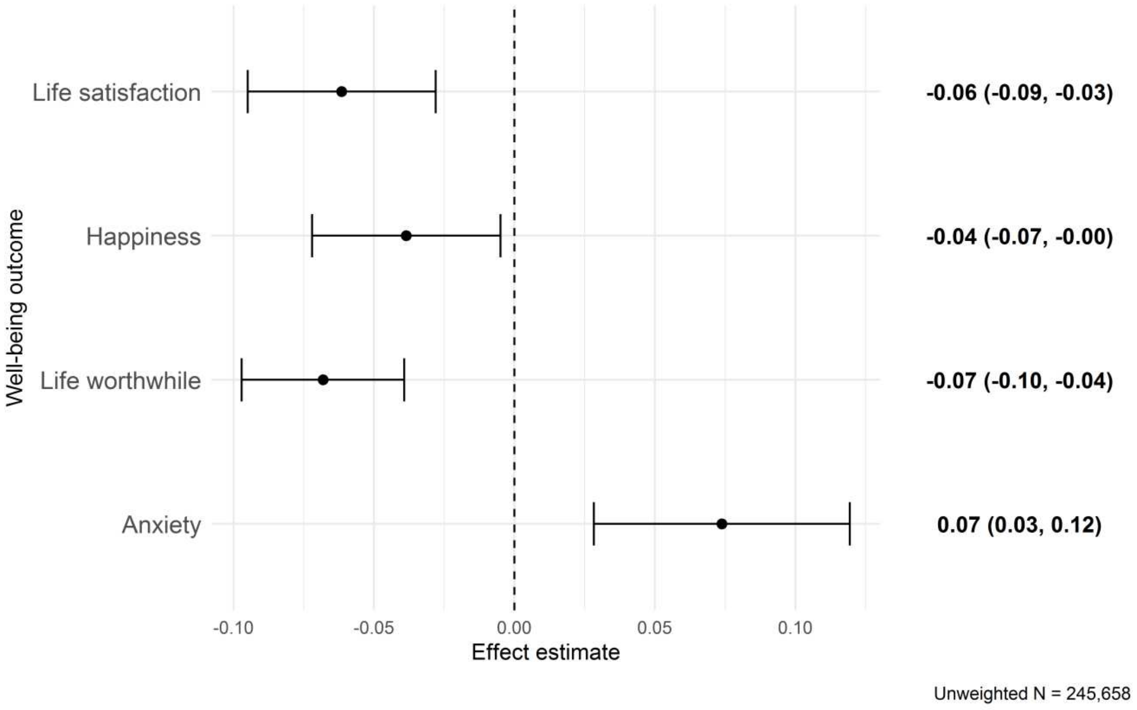

Figure B-11 Well-being effects across UC period (Two-stage model estimations)

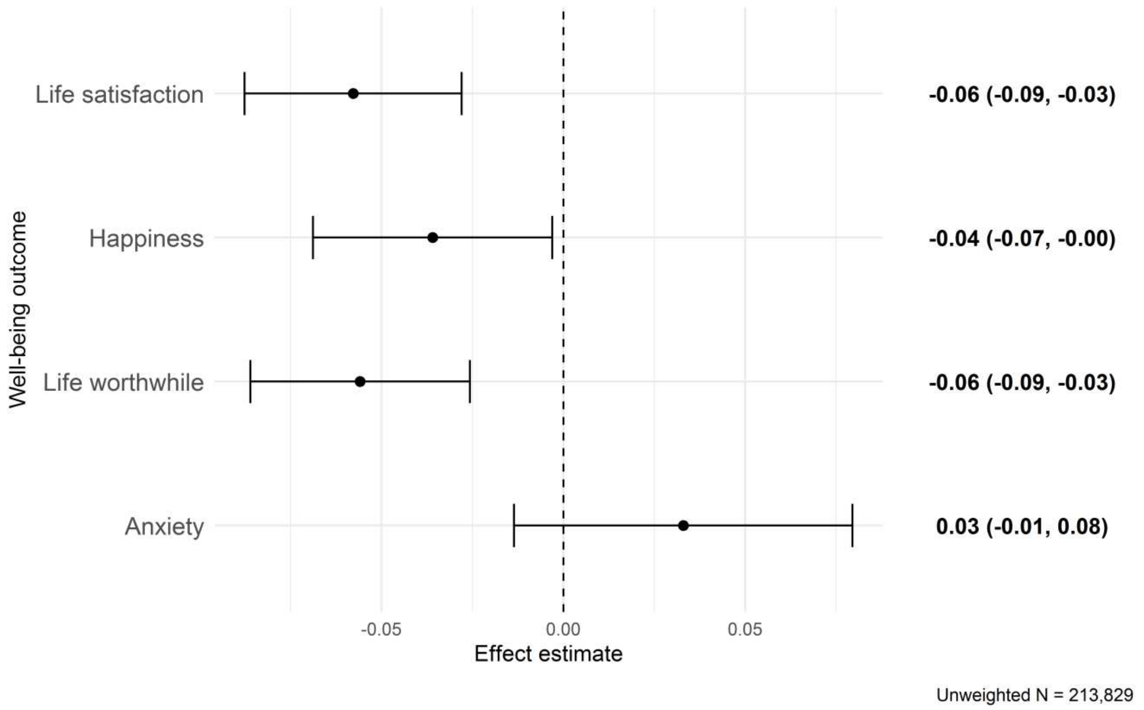

Figure B-12 Wellbeing effects across truncated UC period (2013 to 2-years post-UC; Two-stage model estimations)

Supplement C - Subgroup outcomes

C.1 Relationship status

C.1.1 Subgroup effects

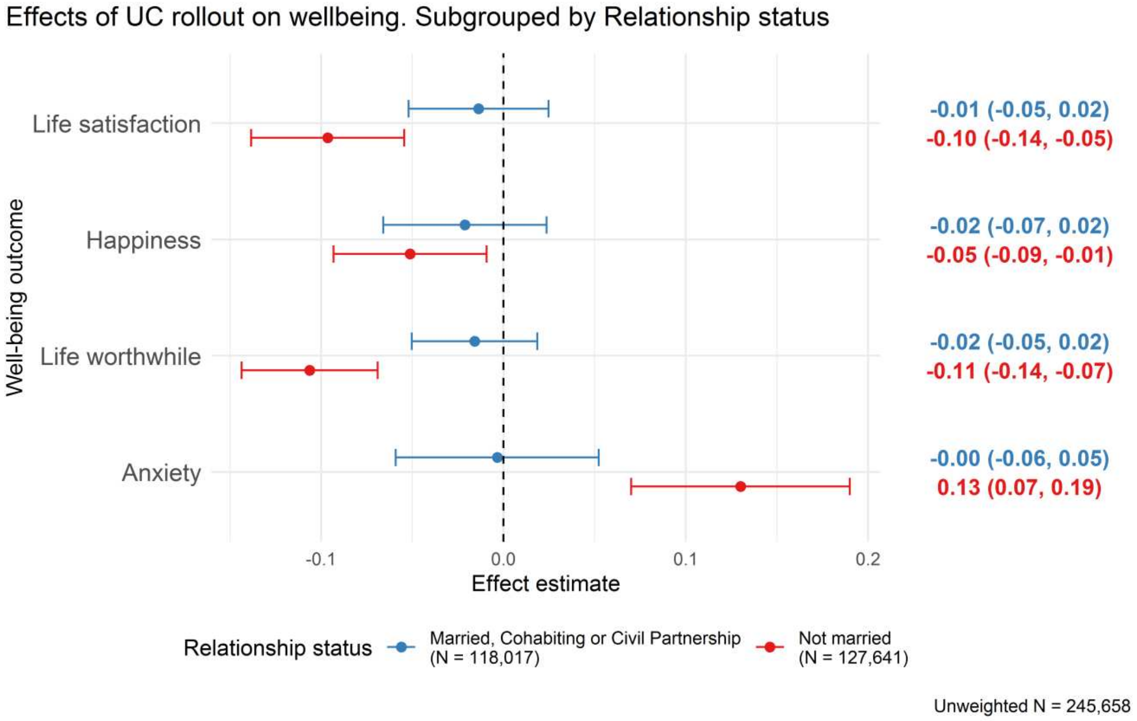

Figure C-1: Within-subgroup effects of Universal Credit on wellbeing; subgroup by Relationship status

C.2 Disability

C.2.1 Subgroup effects

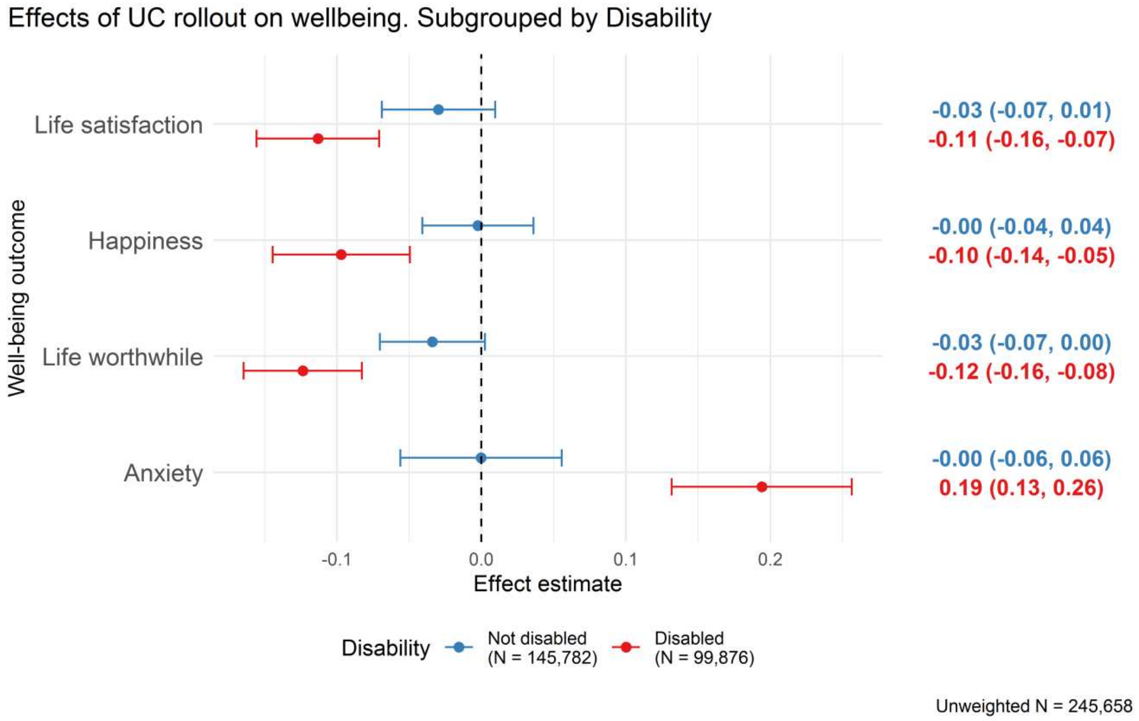

Figure C-2: Within-subgroup effects of Universal Credit on wellbeing; subgroup by Disability

C.3 Aged under 25

C.3.1 Subgroup effects

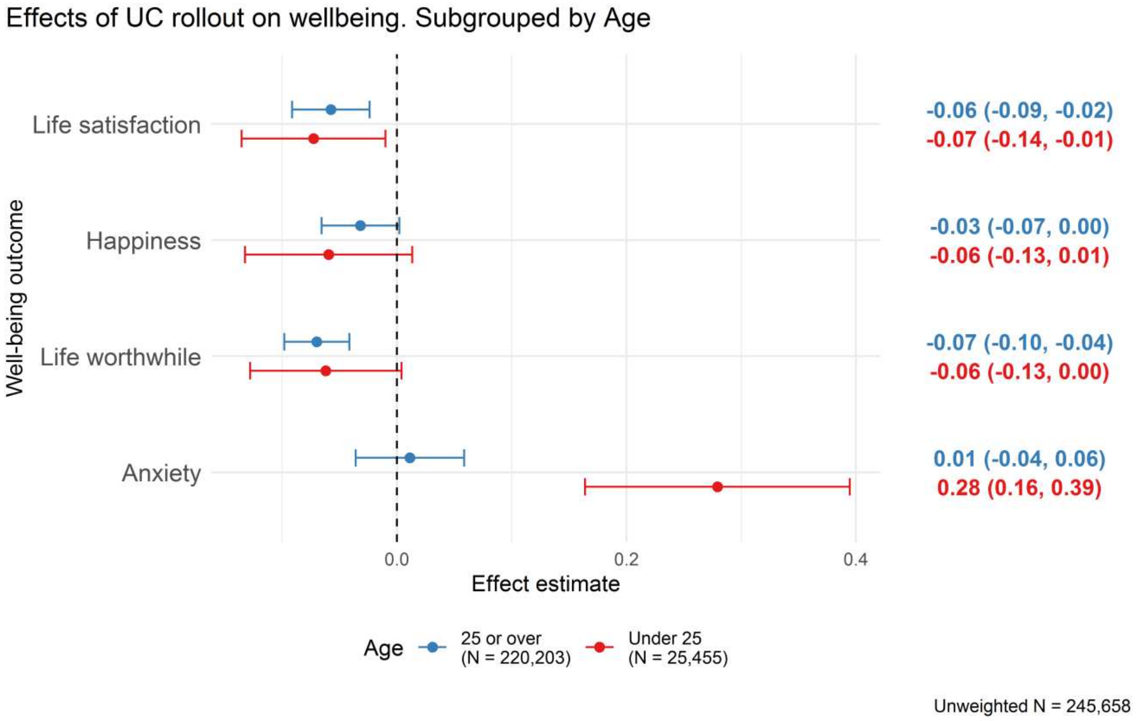

Figure C-3: Within-subgroup effects of Universal Credit on wellbeing; subgroup by Age

C.4 Parent of 1+ children

C.4.1 Subgroup effects

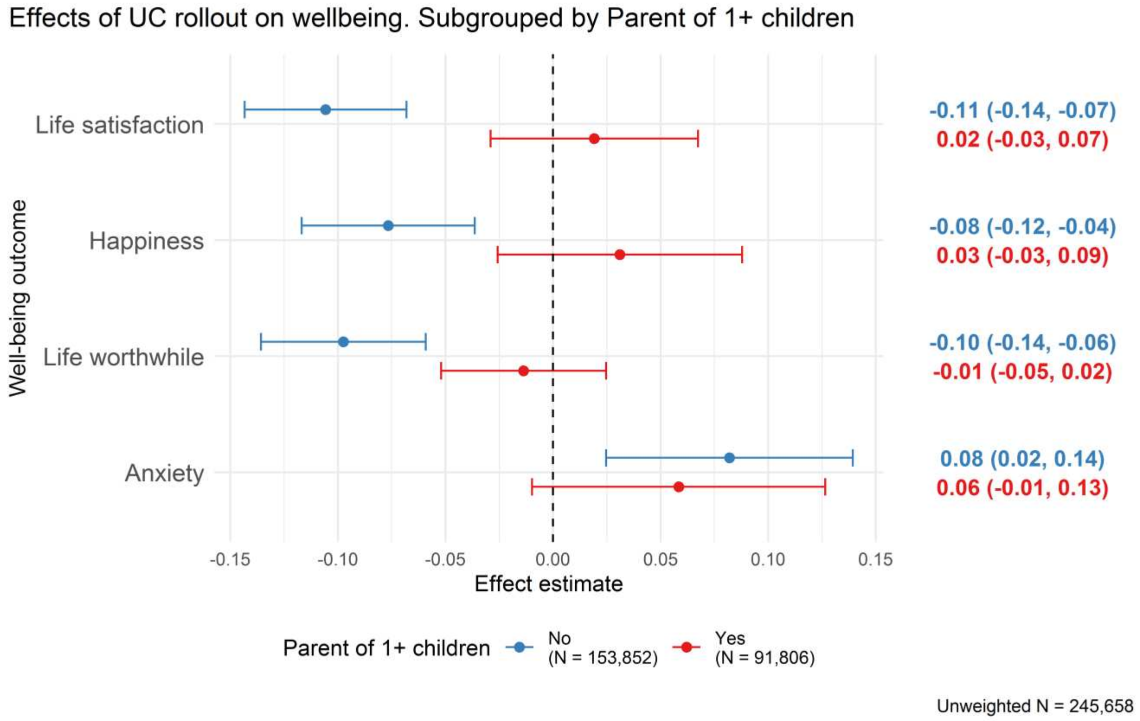

Figure C-4: Within-subgroup effects of Universal Credit on wellbeing; subgroup by Parent of 1+ children

C.5 Caring responsibilities

C.5.1 Subgroup effects

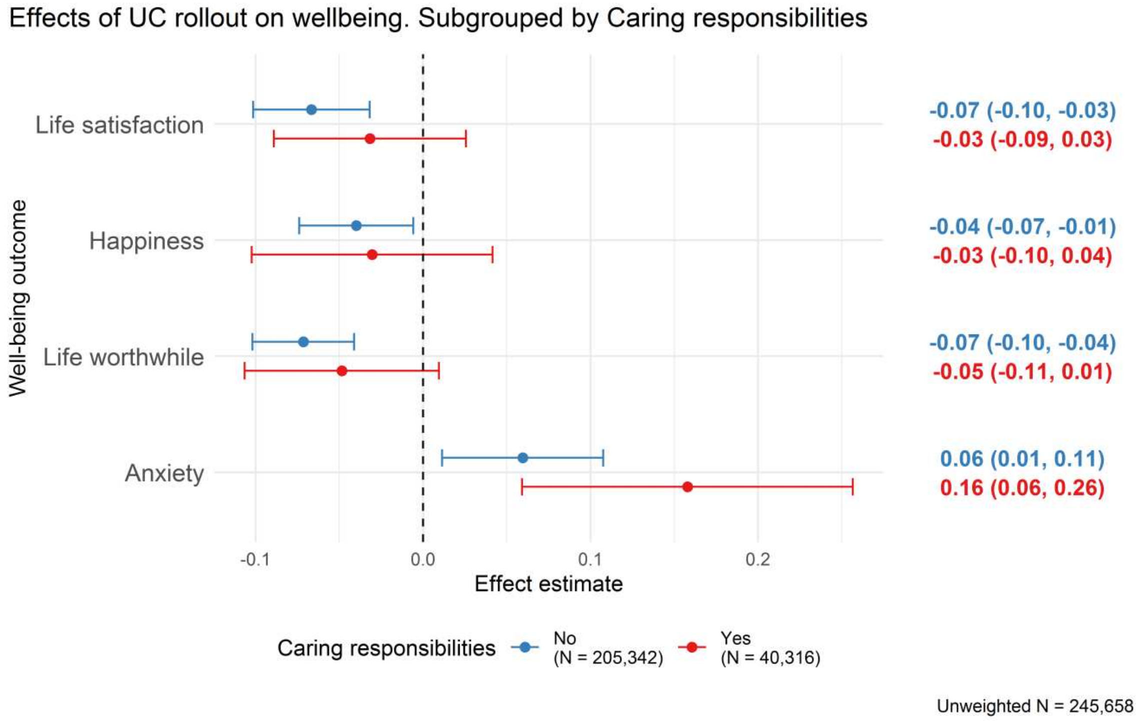

Figure C-5: Within-subgroup effects of Universal Credit on wellbeing; subgroup by Caring responsibilities

C.6 Sex

C.6.1 Subgroup effects

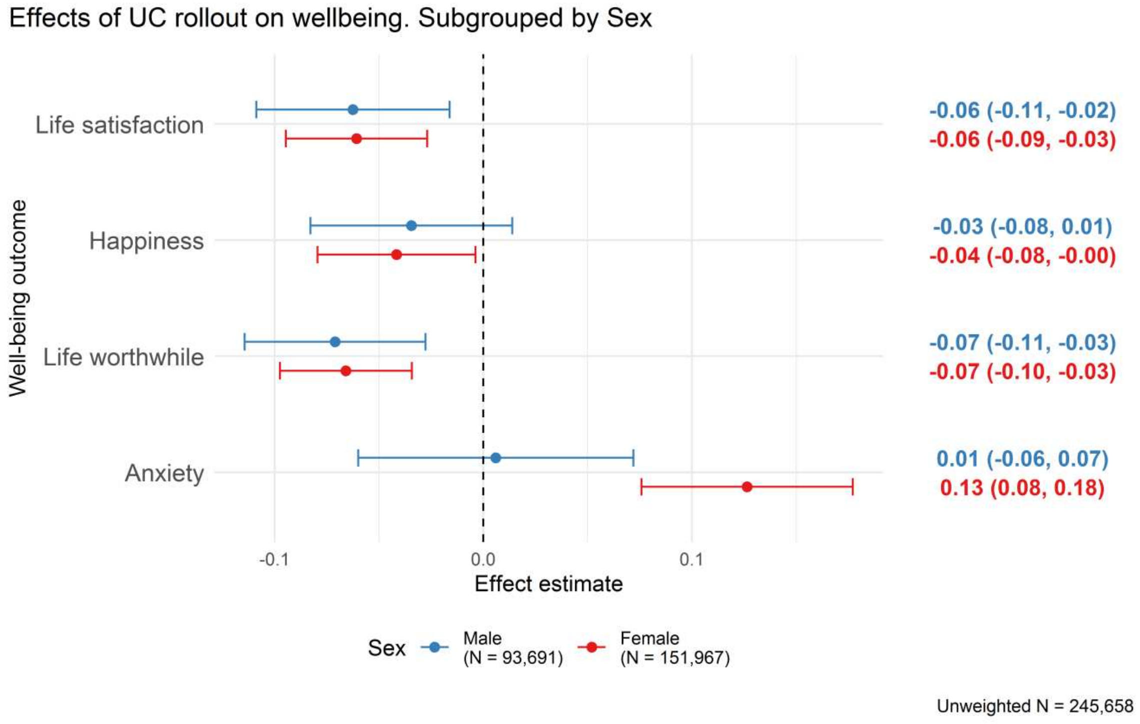

Figure C-6: Within-subgroup effects of Universal Credit on wellbeing; subgroup by Sex

C.7 Country

C.7.1 Subgroup effects

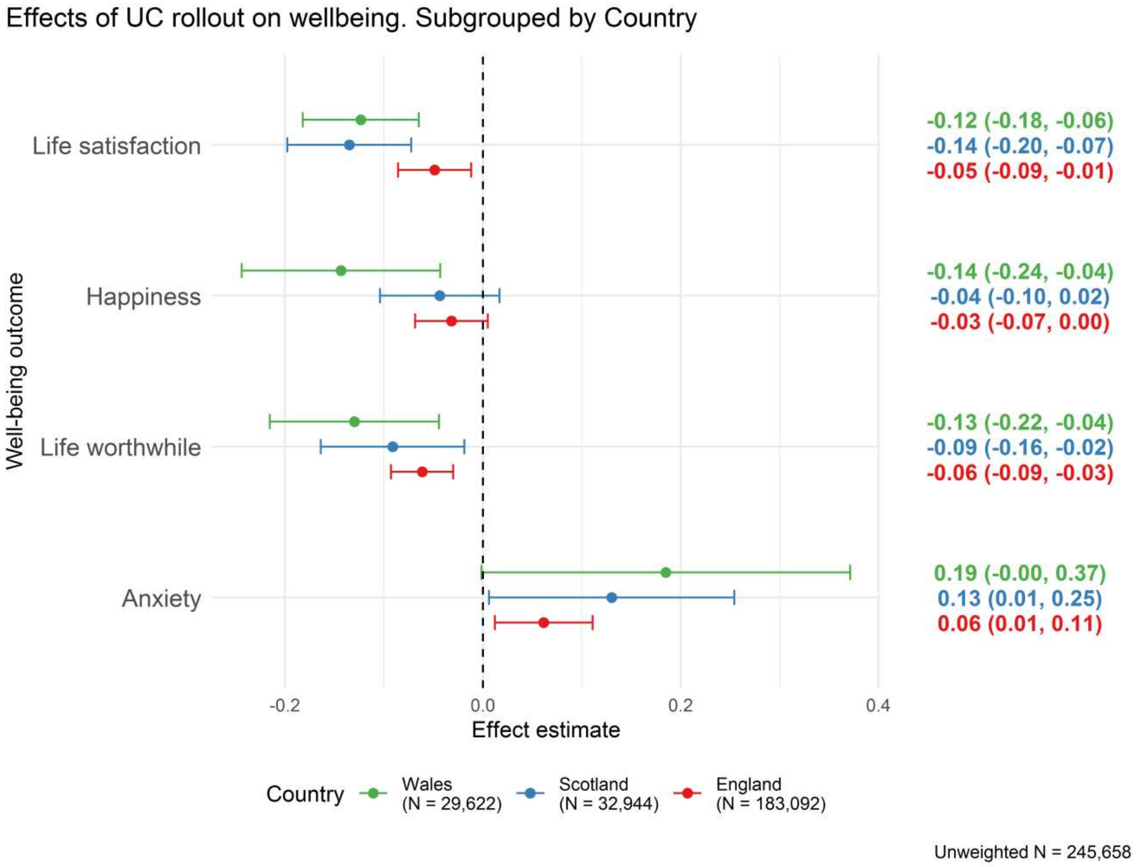

Figure C-7: Within-subgroup effects of Universal Credit on wellbeing; subgroup by Country

C.8 Student status

C.8.1 Subgroup effects

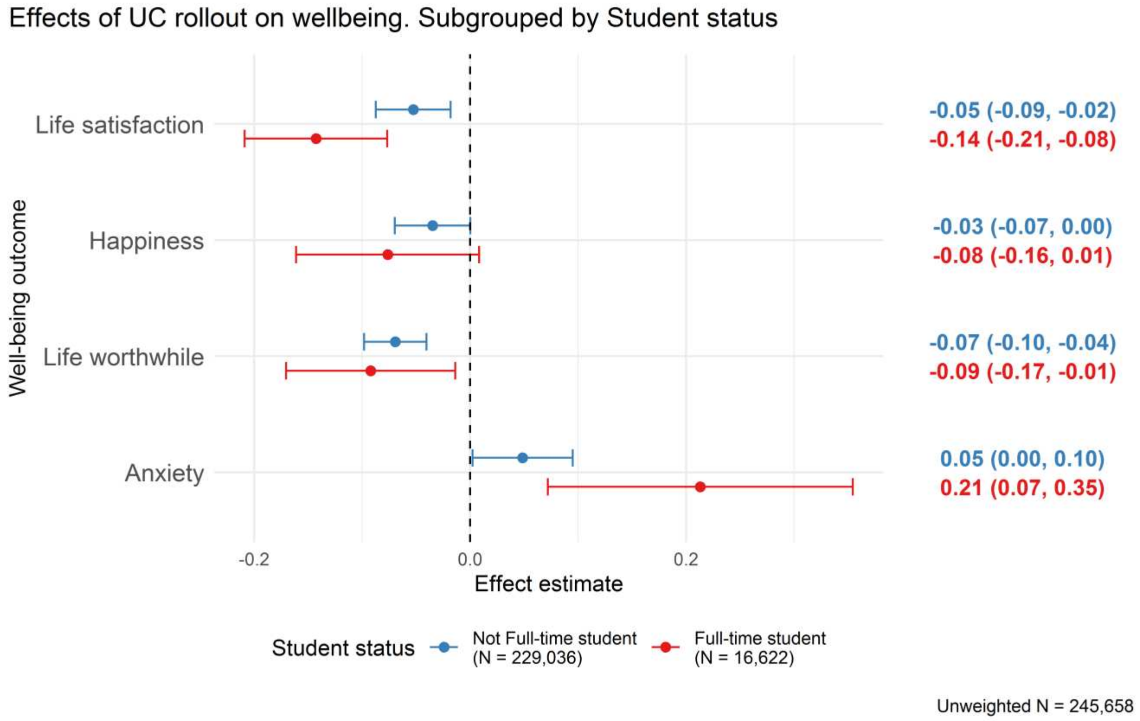

Figure C-8: Within-subgroup effects of Universal Credit on wellbeing; subgroup by Student status

C.9 Ethnicity

C.9.1 Subgroup effects

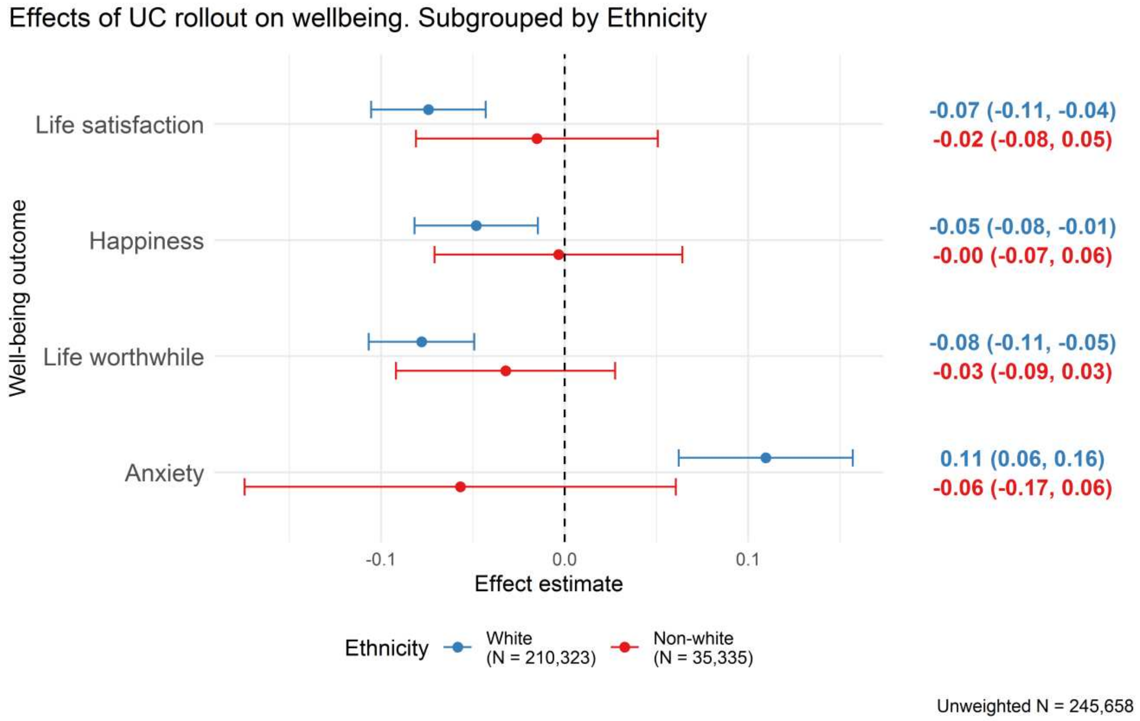

Figure C-9: Within-subgroup effects of Universal Credit on wellbeing; subgroup by Ethnicity

C.10 Education

C.10.1 Subgroup effects

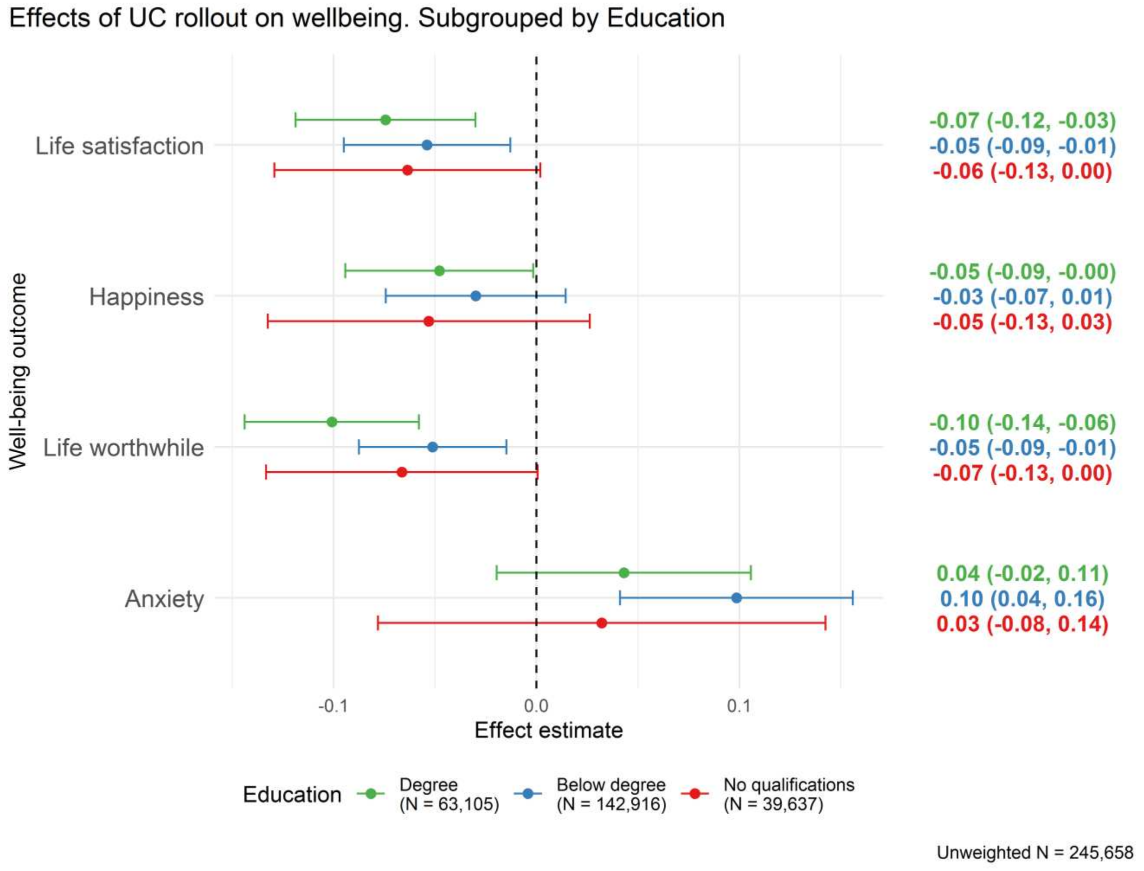

Figure C-10: Within-subgroup effects of Universal Credit on wellbeing; subgroup by Education

C.11 Single parents - 3-way interaction

C.11.1 Subgroup effects

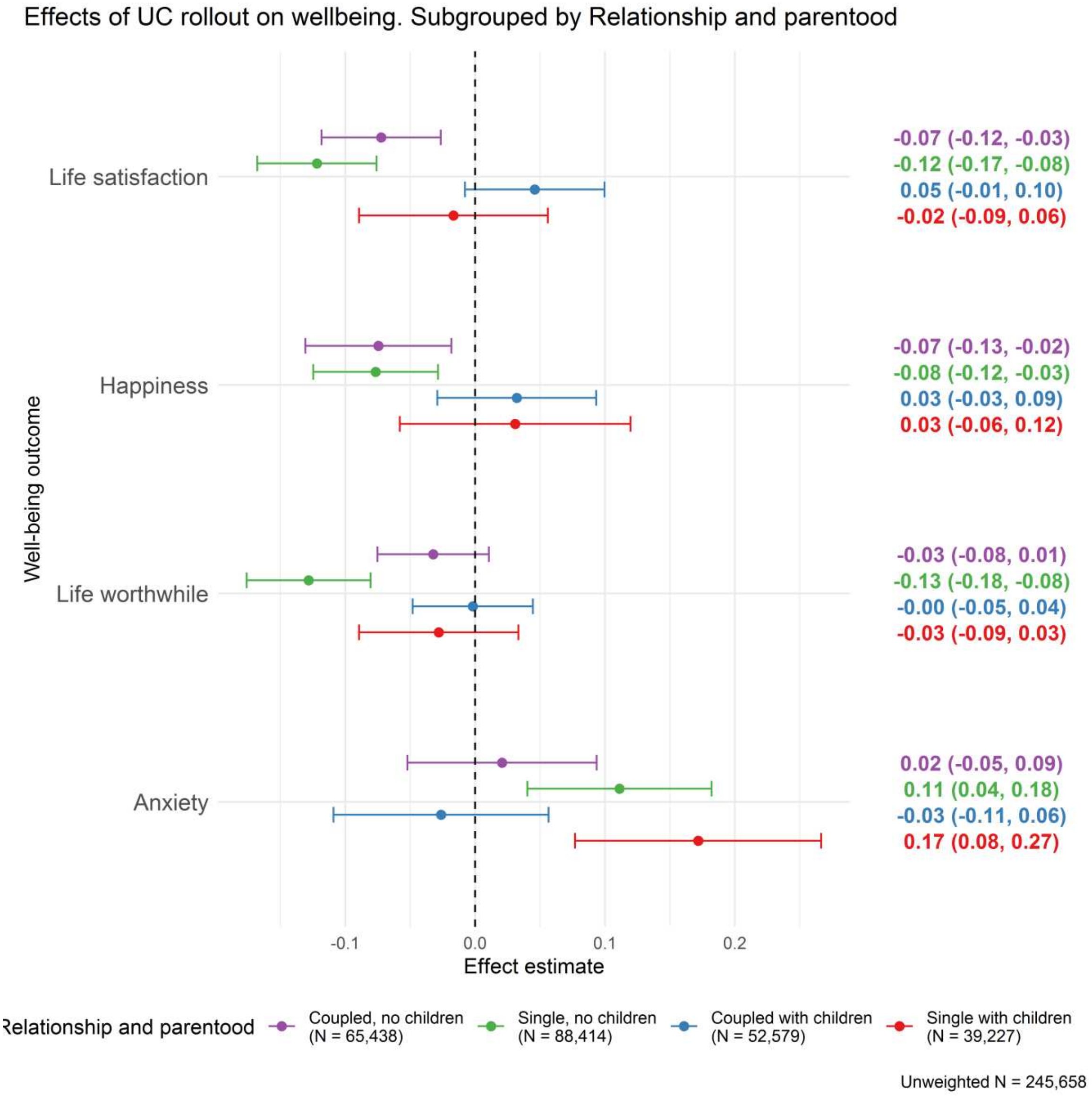

Figure C-11: Within-subgroup effects of Universal Credit on wellbeing; subgroup by Relationship and parenthood

C.12 Overall Interaction effects

Table C-1: Collated interaction effects across all subgroup analyses

	Life satisfaction		Happiness		Life worthwhile		Anxiety	
**Comparison**	**estimate**	**95% CI**	**estimate**	**95% CI**	**estimate**	**95% CI**	**estimate**	**95% CI**
Relationship status								

	Life satisfaction		Happiness		Life worthwhile		Anxiety	
**Comparison**	**estimate**	**95% CI**	**estimate**	**95% CI**	**estimate**	**95% CI**	**estimate**	**95% CI**
‘Not married‘ - ‘Married, Cohabiting or Civil Partnership‘	-0.083	-0.129 to -0.037	-0.030	-0.085 to 0.025	-0.090	-0.135 to -0.046	0.133	0.060 to 0.207
Disability								
‘Disabled‘ - ‘Not disabled‘	-0.083	-0.131 to -0.036	-0.095	-0.147 to -0.042	-0.090	-0.140 to -0.039	0.194	0.118 to 0.271
Aged under 25								
‘Under 25‘ - ‘25 or over‘	-0.015	-0.076 to 0.046	-0.028	-0.101 to 0.046	0.008	-0.058 to 0.074	0.268	0.146 to 0.390
Parent of 1+ children								
‘Yes‘ - ‘No‘	0.125	0.072 to 0.178	0.108	0.039 to 0.176	0.084	0.031 to 0.136	-0.024	-0.110 to 0.062
Caring responsibilities								
‘Yes‘ - ‘No‘	0.035	-0.021 to 0.091	0.009	-0.061 to 0.080	0.023	-0.036 to 0.082	0.098	-0.005 to 0.202
Sex								
‘Female‘ - ‘Male‘	0.002	-0.042 to 0.045	-0.007	-0.061 to 0.046	0.005	-0.042 to 0.052	0.120	0.048 to 0.193
Country								
‘Wales‘ - ‘England‘	-0.075	-0.142 to -0.007	-0.112	-0.216 to -0.007	-0.068	-0.157 to 0.020	0.124	-0.067 to 0.314
‘Scotland‘ - ‘England‘	-0.086	-0.157 to -0.015	-0.012	-0.080 to 0.057	-0.030	-0.108 to 0.048	0.069	-0.067 to 0.204
‘Scotland‘ - ‘Wales‘	-0.012	-0.095 to 0.072	0.100	-0.015 to 0.215	0.039	-0.072 to 0.149	-0.055	-0.277 to 0.167
Student status								
‘Full-time student‘ - ‘Not Full-time student‘	-0.090	-0.158 to -0.022	-0.042	-0.131 to 0.047	-0.023	-0.101 to 0.055	0.165	0.019 to 0.310
Ethnicity								

	Life satisfaction		Happiness		Life worthwhile		Anxiety	
**Comparison**	**estimate**	**95% CI**	**estimate**	**95% CI**	**estimate**	**95% CI**	**estimate**	**95% CI**
'Non-white' - 'White'	0.059	-0.001 to 0.119	0.045	-0.022 to 0.112	0.046	-0.012 to 0.104	-0.166	-0.295 to -0.038
Education								
'Below degree' - 'Degree'	0.020	-0.026 to 0.067	0.018	-0.038 to 0.074	0.050	0.001 to 0.098	0.056	-0.020 to 0.131
'No qualifications' - 'Degree'	0.011	-0.062 to 0.083	-0.005	-0.094 to 0.084	0.035	-0.046 to 0.115	-0.011	-0.130 to 0.108
'No qualifications' - 'Below degree'	-0.010	-0.084 to 0.065	-0.023	-0.114 to 0.068	-0.015	-0.088 to 0.058	-0.066	-0.186 to 0.053
Relationship and parenthood								
'Single, no children' - 'Coupled, no children'	-0.049	-0.106 to 0.007	-0.002	-0.069 to 0.064	-0.096	-0.152 to -0.040	0.090	-0.001 to 0.182
'Coupled with children' - 'Coupled, no children'	0.118	0.055 to 0.182	0.107	0.030 to 0.183	0.031	-0.026 to 0.087	-0.047	-0.155 to 0.061
'Single with children' - 'Coupled, no children'	0.056	-0.026 to 0.138	0.105	-0.005 to 0.216	0.004	-0.073 to 0.081	0.151	0.028 to 0.274
'Coupled with children' - 'Single, no children'	0.168	0.104 to 0.232	0.109	0.034 to 0.183	0.126	0.065 to 0.188	-0.137	-0.242 to -0.033
'Single with children' - 'Single, no children'	0.105	0.026 to 0.184	0.107	0.005 to 0.210	0.100	0.020 to 0.181	0.061	-0.053 to 0.174
'Single with children' - 'Coupled with children'	-0.063	-0.143 to 0.017	-0.001	-0.098 to 0.095	-0.026	-0.100 to 0.048	0.198	0.086 to 0.310

Supplement D - Sensitivity analysis – population observed receiving benefits

Level	Removed	Population
All observations	-	921,139
No missing data	14,745	906,394
Sufficient pre-post data	8,290	898,104
Over 18 and pre-2020	83,317	814,787
Report claiming UC or Legacy Benefit	624,943	189,844

Table D-1 – Population inclusion by criteria

An average of 12.2% (N = 2574) of respondents in benefit claiming households reported receiving Universal Credit across the first year following UC rollout.

18.1% (N = 5792) reported receiving UC across all years where UC was available in their Local Authority (85.9%, N = 33027 claimed legacy benefits).

At 1 year, 21.3% (N = 872) of surveyed respondents in benefit claiming households reported receiving UC. At 2 years, 29.5% (N = 466) reported receiving UC.

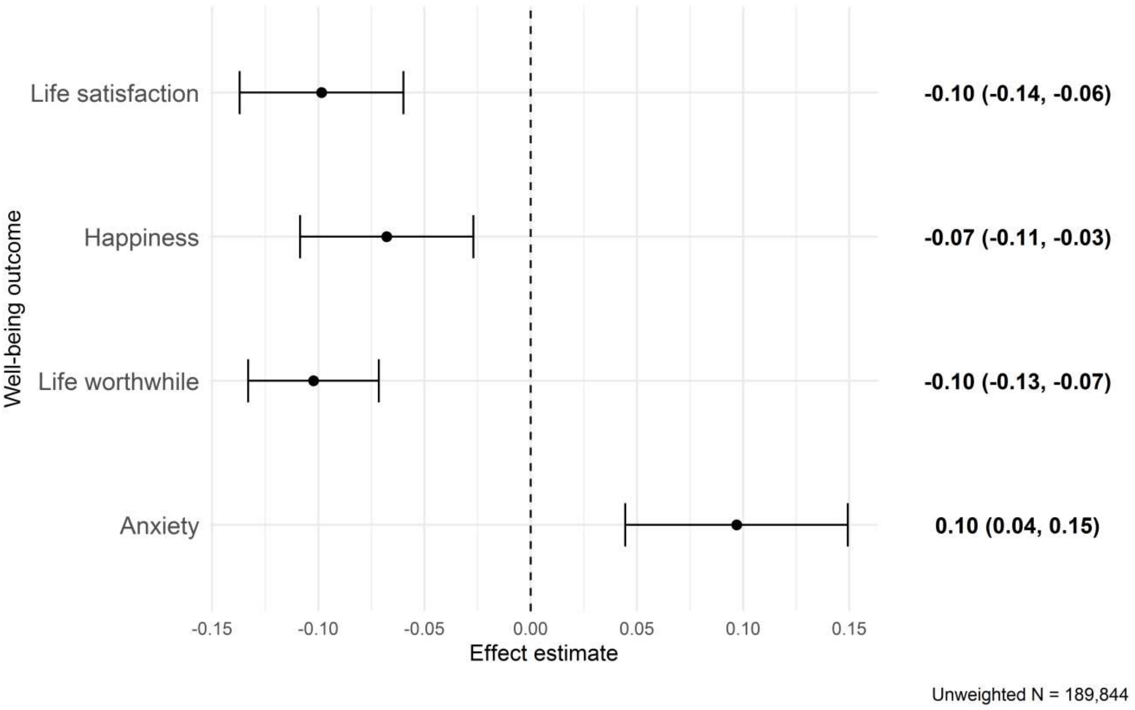

Figure D-1 Well-being effects across UC period (Two-stage model estimations)

Supplement E - Sensitivity analyses – differing wage thresholds

E.1 Income across populations

	10th percentile	Lower quartile	Median	Upper quartile	90th percentile
**Received UC**	**Income**	**Income**	**Income**	**Income**	**Income**
No	£580	£1,100	£1,830	£2,730	£3,820
Yes	£240	£390	£670	£1,070	£1,550
Unweighted N = 543,195					

Table E-1 Median, and quantiles of monthly equivalised income across households receiving and not receiving Universal Credit or a legacy benefit (rounded to the nearest £10)

The median wage of UC claimants was £8,040 per year and the 90th percentile was £18,600 per year. These were selected as higher and lower thresholds than the previously tested 75th percentile for sensitivity testing.

E.2 Population earning less than £8,040 per year

E.2.1 Exposure in post-UC period

Population = 222,492 (unweighted individuals). Weighted observations: 80,168,274 - living in households with equivalised household income < £8,040pa and not retired.

Figure E-1 shows the percentage of low-income respondents in each period relative to the rollout of UC who report claiming Universal Credit.

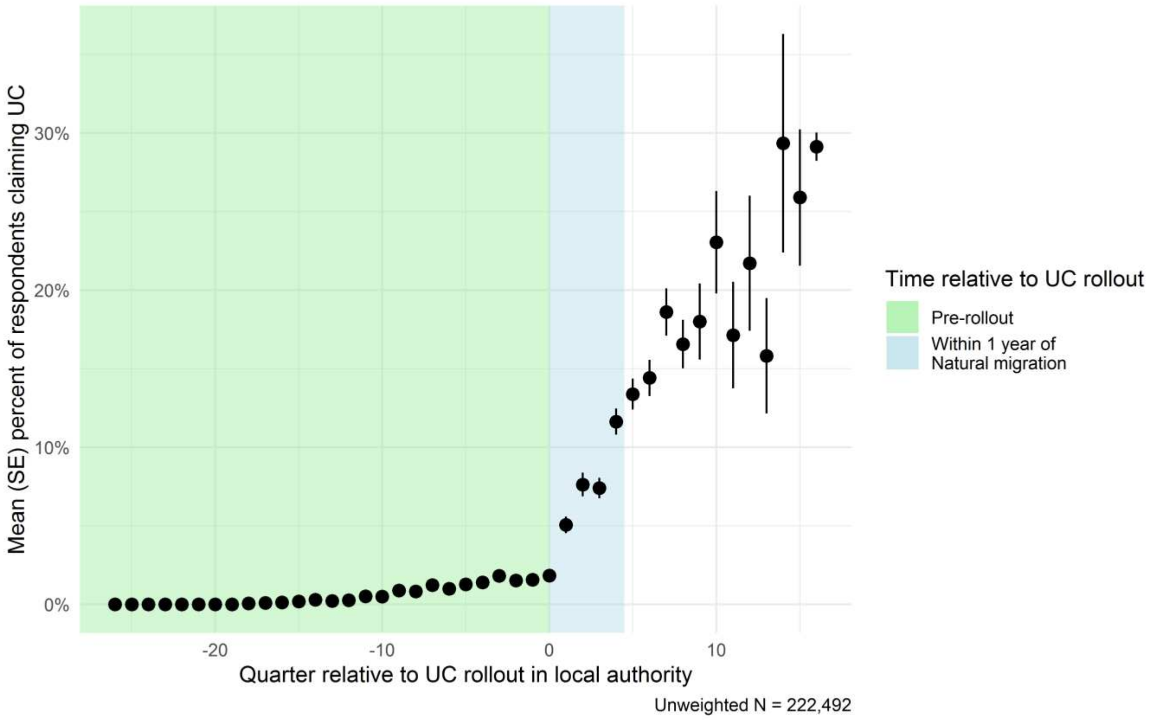

Figure E-1 Percentage of respondents per Local Authority reporting claiming UC in each quarter relative to UC rollout in their area.

9.9% (N = 4478) reported receiving UC across all years where UC was available in their Local Authority (39.5%, N = 21298 claimed legacy benefits).

E.2.2 Two-stage DiD models

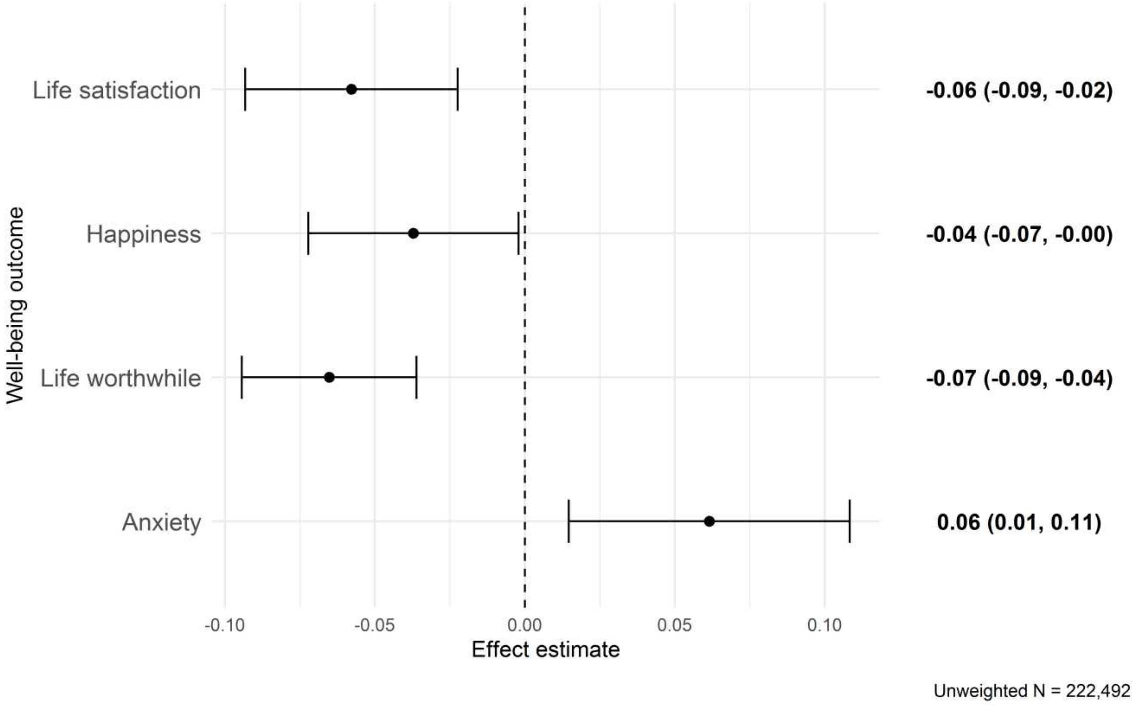

Figure E-2 Well-being effects across UC period (Two-stage model estimations)

E.3 Population earning less than £18,600 per year

E.3.1 Exposure in post-UC period

Population = 444,069 (unweighted individuals). Weighted observations: 158,658,923 - living in households with equivalised household income < £18,600pa and not retired.

Figure E-3 shows the percentage of low-income respondents in each period relative to the rollout of UC who report claiming Universal Credit.

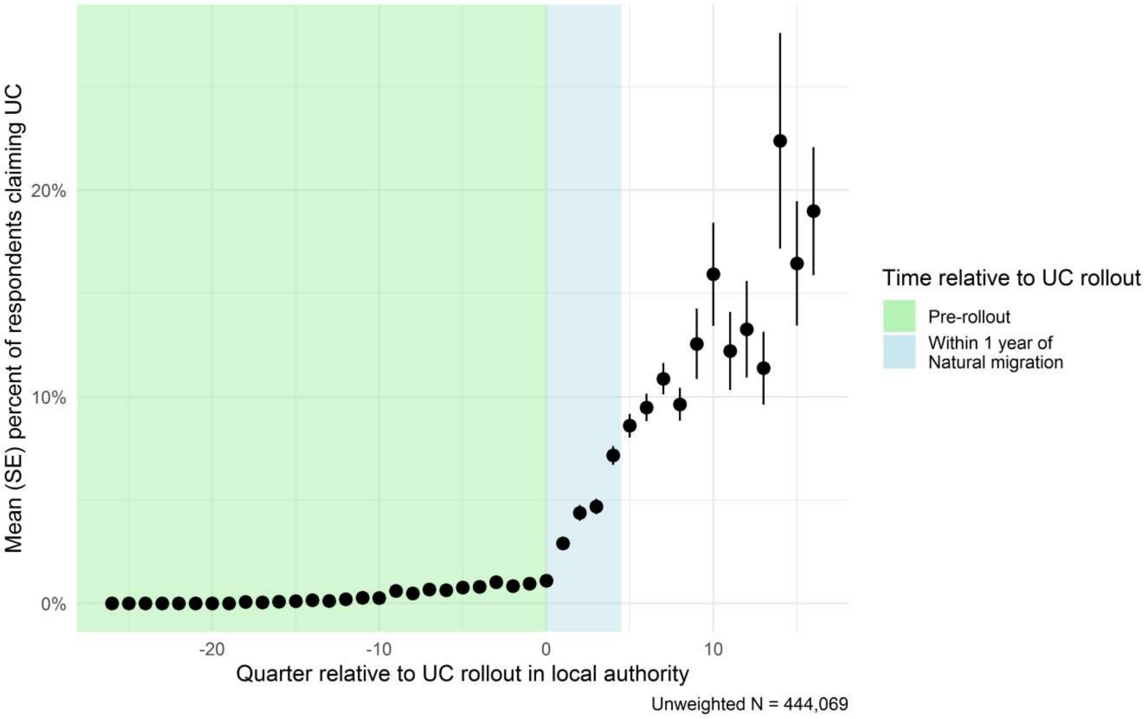

Figure E-3 Percentage of respondents per Local Authority reporting claiming UC in each quarter relative to UC rollout in their area.

6.2% (N = 5584) reported receiving UC across all years where UC was available in their Local Authority (27.6%, N = 29780 claimed legacy benefits).

E.3.2 Two-stage DiD models

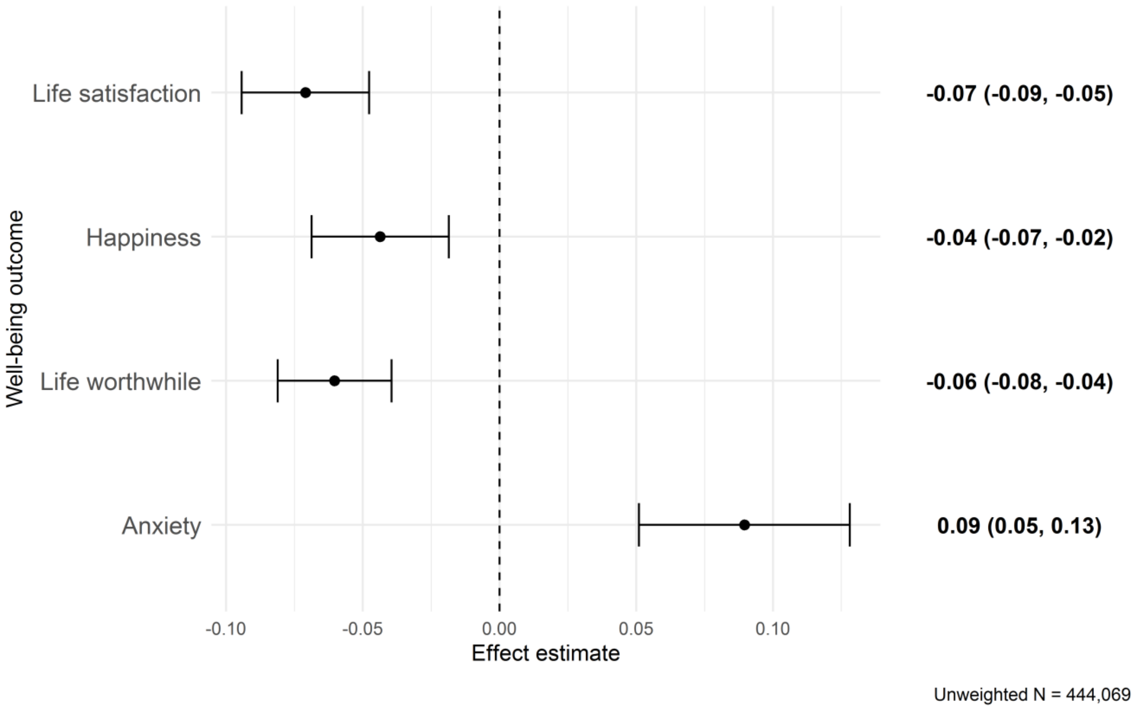

Figure E-4 Well-being effects across UC period (Two-stage model estimations)

Supplement F - Deviations from analysis protocol

The analysis plan was published alongside other study documentation at https://osf.io/knajb/.

The following deviations from the overall protocol are noted:

- Research questions looking at mediating factors of changes in employment and income were not addressed in this analysis (p7)
- Two methods for determining eligible population were described in the protocol: marking all benefit receipt as criteria for inclusion and predicting probability of receipt using a statistical model trained on observed data (p11). These were deemed insufficient for having necessary specificity and sensitivity and creating comparable populations across states of rollout. They also made effect estimates hard to interpret. We opted to set an equivalised income threshold as a marker of inclusion as a more manageable method and use observed benefit receipt as a marker in sensitivity analyses.
- A sensitivity analysis treating restricted-rollout-eligible respondents in the pre-natural-migration period as 'exposed' was not carried out. We did not have sufficient data to reliably identify this sub-population (p27).

- In subgroup analyses:
    - We did not test for differential effects by pre-UC employment. This data was not available for almost all participants.
    - We added education as a marker of vulnerability to test over.
    - We had specified running stratified subgroup analyses alongside interaction models. To produce estimates of subgroups which were comparable with interaction effects we calculated stratified differences in one model per subgroup and compared these using the ‘marginaleffects‘ package.
- We added a set of area-wide confounder variables to better account for wider determinants of health not captured in individual responses (p19)
- Missing data was deemed to be sufficiently low that multiple imputation was not needed over complete-case analysis (p23)
- In fitting models to account for effect heterogeneity, we were unable to generate effect estimates using the ‘did‘ package or the other suggested methods. Several of these are built for balanced panel data and did not function for our cross-sectional dataset (p24).
